# The Endosomal–Lysosomal Pathway Is Dysregulated by *APOE4* Expression *in Vivo*

**DOI:** 10.3389/fnins.2017.00702

**Published:** 2017-12-12

**Authors:** Tal Nuriel, Katherine Y. Peng, Archana Ashok, Allissa A. Dillman, Helen Y. Figueroa, Justin Apuzzo, Jayanth Ambat, Efrat Levy, Mark R. Cookson, Paul M. Mathews, Karen E. Duff

**Affiliations:** ^1^Department of Pathology and Cell Biology, Taub Institute for Research of Alzheimer's Disease and the Aging Brain, Columbia University Medical Center, Colombia University, New York, NY, United States; ^2^Department of Neuroscience and Physiology, New York University Langone Medical Center, New York University, New York, NY, United States; ^3^Cell Biology and Gene Expression Section, Laboratory of Neurogenetics, National Institute on Aging, National Institutes of Health, Bethesda, MD, United States; ^4^Laboratory of Receptor Biology and Gene Expression, Center for Cancer Research, National Cancer Institute, National Institutes of Health, Bethesda, MD, United States; ^5^Center for Dementia Research, Nathan S. Kline Institute, Orangeburg, NY, United States; ^6^Department of Biochemistry and Molecular Pharmacology, New York University Langone Medical Center, New York University, New York, NY, United States; ^7^Neuroscience Institute, New York University Langone Medical Center, New York University, New York, NY, United States; ^8^Department of Psychiatry, New York University Langone Medical Center, New York University, New York, NY, United States; ^9^Division of Integrative Neuroscience in the Department of Psychiatry, New York State Psychiatric Institute, New York, NY, United States

**Keywords:** Apolipoprotein E, *APOE*, *APOE4*, endosome, lysosome, Alzheimer's disease, transcriptomics, RNA-seq

## Abstract

Possession of the ε4 allele of apolipoprotein E (*APOE*) is the major genetic risk factor for late-onset Alzheimer's disease (AD). Although numerous hypotheses have been proposed, the precise cause of this increased AD risk is not yet known. In order to gain a more comprehensive understanding of *APOE4*'s role in AD, we performed RNA-sequencing on an AD-vulnerable vs. an AD-resistant brain region from aged APOE targeted replacement mice. This transcriptomics analysis revealed a significant enrichment of genes involved in endosomal–lysosomal processing, suggesting an *APOE4*-specific endosomal–lysosomal pathway dysregulation in the brains of *APOE4* mice. Further analysis revealed clear differences in the morphology of endosomal–lysosomal compartments, including an age-dependent increase in the number and size of early endosomes in *APOE4* mice. These findings directly link the *APOE4* genotype to endosomal–lysosomal dysregulation in an *in vivo*, AD pathology-free setting, which may play a causative role in the increased incidence of AD among *APOE4* carriers.

## Introduction

Apolipoprotein E (*APOE*) plays a vital role in the transport of cholesterol and other lipids through the bloodstream, as well as within the brain (Mahley and Rall, 2000; Han, 2004; Holtzman et al., 2012). In addition, possession of the ε4 allele of *APOE* is the primary genetic risk factor for developing late-onset Alzheimer's disease (AD). While *APOE4*'s role in AD has often been attributed to its ability to increase the aggregation and decrease the clearance of Aβ (Rebeck et al., 1993; Schmechel et al., 1993; Ma et al., 1994; Castano et al., 1995; Bales et al., 1997; Holtzman et al., 2000; Castellano et al., 2011), *APOE4* expression has also been shown to affect a wide array of Aβ-independent processes in the brain [see reviews by Huang (2010) and Wolf et al. (2013)], including a potential role in increasing the pathogenicity of other proteins, such as tau and α-synuclein (see review by Huynh et al., 2017).

Given these observations, it is important to elucidate the full range of effects that *APOE4* expression has on the brain in order to identify new mechanisms that might be responsible for the increased AD risk among *APOE4* carriers. To that end, we have performed RNA-sequencing on the entorhinal cortex (EC) and the primary visual cortex (PVC) from 14 to 15 month old *APOE3/4* vs. *APOE3/3* targeted replacement mice. *APOE* targeted replacement mice express human *APOE* in place of the mouse *Apoe* gene and are devoid of overt AD pathology (Sullivan et al., 1997, 2004). Since the majority of *APOE4* carriers possess only one ε4 allele (~22.2 vs. ~1.9% with two alleles, according to AlzGene), we chose to limit our initial comparison to mice expressing *APOE3/4* vs. *APOE3/3*. The EC was utilized as an AD-vulnerable brain region because it is one of the first brain regions to develop AD pathology (Braak and Braak, 1991; Bobinski et al., 1999), and it has been shown to be particularly vulnerable to *APOE4*-linked morphological changes (Shaw et al., 2007; Rodriguez et al., 2013; DiBattista et al., 2014). The PVC was utilized as an AD-resistant brain region because it is relatively spared in AD (Braak and Braak, 1991; Minoshima et al., 1997; Wang et al., 2010) and it has not been reported to demonstrate any *APOE4*-linked morphological changes.

Our transcriptomics analysis identified over 1,000 genes that were differentially affected by *APOE4* gene expression in the EC. Endosomal–lysosomal pathway genes were significantly enriched among these genes. To confirm the biological importance of these gene expression differences, we examined brain sections by immunohistochemistry from *APOE4/4* vs. *APOE3/3* mice, which revealed morphological differences in endosomal–lysosomal compartments in the *APOE4/4* mice, including an age-dependent increase in the number and size of early endosomes in the brains of these mice. Together, these findings implicate *APOE4* in the dysregulation of the endosomal–lysosomal pathway in the brain, which may be a contributing factor to the development of AD among *APOE4* carriers.

## Materials and methods

### Mice

All experimental procedures involving animals in this study were approved by and complied with the guidelines of the Institutional Animal Care and Use Committee of the Nathan Kline Institute and Columbia University Medical Center. Human *APOE* targeted replacement mice were originally developed by Sullivan et al. (1997, 2004). These targeted-replacement mice express human *APOE* under the control of the endogenous murine promoter, which allows for the expression of human *APOE* at physiologically regulated levels in the same temporal and spatial pattern as endogenous murine *Apoe*. For our initial transcriptomics analysis, 14–15 month old male *APOE3/4* vs. *APOE3/3* mice were used, whereas immunohistochemical analysis of endosomal and lysosomal morphology was performed on approximately equal numbers of male and female *APOE4/4* vs. *APOE3/3* mice at 6, 12, 18, and 25 months of age. Mice were derived from two separate colonies. The *APOE* genotype of the mice was confirmed either in-house by restriction fragment length polymorphism analysis following polymerase chain reaction (PCR), as previously described (Hixson and Vernier, 1990) or using a PCR-based method developed by Transnetyx (Cordova, TN).

### RNA extraction for transcriptomics analysis

Mouse numbers were selected to be similar to those used in previous experiments (Hauser et al., 2014; Dillman et al., 2016). Male mice expressing human *APOE3/3* (10 mice) or *APOE3/4* (19 mice) were aged to 14–15 months, at which point they were sacrificed by cervical dislocation, and brain tissues containing the EC and PVC were dissected and snap frozen on dry-ice. Brain tissues were stored in RNase-free eppendorf tubes at −80°C prior to extraction. Total RNA was extracted from frozen tissues by homogenizing each tissue sample using a battery-operated pestle mixer (Argos Technologies, Vernon Hills, IL) in 1 ml of TRIzol reagent according to the manufacturer's protocol (Life Technologies, Carlsbad, CA). RNA concentration was measured using a nanodrop 1000 (Thermo Fisher Scientific, Waltham, MA), and RNA integrity (RIN) was assessed using an Agilent 2100 Bioanalyzer (Agilent Technologies, Santa Clara, CA). All RNA samples possessed a RIN of 8 or higher. RNA was stored at −80°C prior to use.

### RNA-sequencing and analysis

Starting with 2 μg of total RNA per sample, Poly(A)+ mRNA was purified, fragmented and then converted into cDNA using the TruSeq RNA Sample Prep Kit v2 (Illumina cat# RS-122-2001) as per the manufacturer's protocol (Illumina, San Diego, CA). For RNA-Sequencing of the cDNA, we hybridized 5 pM of each library to a flow cell, with a single lane for each sample, and we used an Illumina cluster station for cluster generation. We then generated 149 bp single end sequences using an Illumina HiSeq 2000 sequencer. For analysis, we used the standard Illumina pipeline with default options to analyze the images and extract base calls in order to generate fastq files. We then aligned the fastq files to the mm9 mouse reference genome using Tophat (v2.0.6) and Bowtie (v2.0.2.0). In order to annotate and quantify the reads to specific genes, we used the Python module HT-SEQ with a modified NCBIM37.61 (containing only protein coding genes) gtf to provide reference boundaries. We used the R/Bioconductor package DESeq2 (v1.10.1) for comparison of aligned reads across the samples. We conducted a variance stabilizing transformation on the aligned and aggregated counts, and then the Poisson distributions of normalized counts for each transcript were compared across *APOE3/4* vs. *APOE3/3* groups using a negative binomial test. We corrected for multiple testing using the Benjamini-Hochberg procedure and selected all genes that possessed a corrected *p*-value of less than 0.05. Finally, a heat map based on sample distance and a volcano plot based on fold change and adjusted *p*-values were generated using the R/Bioconductor package ggplot2 (v2.0.0).

### Pathway analysis

Enriched KEGG pathways were identified using the ClueGo application (version 2.1.7) in Cytoscape (version 3.2.1). Briefly, all differentially expressed EC genes from the RNA-Seq analysis were entered into the application and searched for significantly enriched KEGG pathways possessing a Benjamini-Hochberg adjusted *p*-value of less than 0.05. For gene ontology (GO) pathway analysis, differentially expressed EC genes were entered into NIH's DAVID program (https://david.ncifcrf.gov/; version 6.8), and a functional annotation chart was generated for all significantly enriched GO Biological Process Terms, GO Cellular Component Terms and GO Molecular Function Terms possessing a Benjamini-Hochberg adjusted *p*-value of less than 0.05.

### Immunolabeling of fixed brain tissue

For immunolabeling of brain tissue, mice were transcardially perfusion-fixed with 4% paraformaldehyde in phosphate buffered saline (PBS). Brains were removed and post-fixed for 3 days in 4% paraformaldehyde in PBS at 4°C, and subsequently cut into 40 μm-thick coronal sections using a vibratome, as we have described previously (Choi et al., 2013). Free-floating sections were labeled with anti-Rab5a (sc-309; Santa Cruz Biotechnology, Santa Cruz, CA; dilution 1: 100) or an in-house anti-CatD rabbit polyclonal antibody (Yang et al., 2014). Tissue sections were also co-labeled with a Nissl stain to determine the border of the cell soma. Following binding of appropriate fluorophore-conjugated secondary antibodies (Invitrogen, Grand Island, NY; dilution 1:500), immunofluorescence was captured at 100 × magnification using an Axiovert 200M Fluorescence Imaging Microscope (Zeiss, Thornwood, NY) by a genotype-blinded observer. Quantification and morphometric analysis of labeled particles per cell was measured using ImageJ's automated particle analysis (NIH, Bethesda, Maryland) after thresholding the density of Rab5a or CatD-positive signal over background, as we have done previously (Choi et al., 2013). With this method, we obtained automated calculations of average area, particle count, and area fraction within a manually drawn border around the cell soma. Statistical analysis was performed using a Student's *t*-test between groups. Cell and mouse numbers were selected to be similar to those used in previous experiments (Choi et al., 2013; Jiang et al., 2016). For the quantifications in EC, we used *n* = 118 neurons from 7 mice (*APOE3/3*) and *n* = 85 neurons from 6 mice (*APOE4/4*) for Rab5a, and *n* = 126 neurons from 7 mice (*APOE3/3*) and *n* = 139 neurons from 7 mice (*APOE4/4*) for CatD. For the quantifications in cingulate cortex, we used *n* = 48 neurons (*APOE3/3*) and 52 neurons (*APOE4/4*) from 4 mice per genotype at age 6 months, *n* = 115 neurons (*APOE3/*3) and 88 neurons (*APOE4/4*) from 8 mice per genotype at age 12 months, *n* = 267 neurons (*APOE3/3*) and 286 neurons (*APOE4/4*) from 8 mice per genotype at age 18 months, and *n* = 342 neurons (*APOE3/3*) and 141 neurons (*APOE4/4*) from 8 mice per genotype at age 25 months.

### Data availability

The transcriptomics datasets generated during the current study are available in the NCBI Gene Expression Omnibus (GEO) repository, accession # GSE102334.

## Results

### Altered gene expression is observed in the EC and PVC of aged *APOE3/4* vs. *APOE3/3* mice

**Figure 1:**
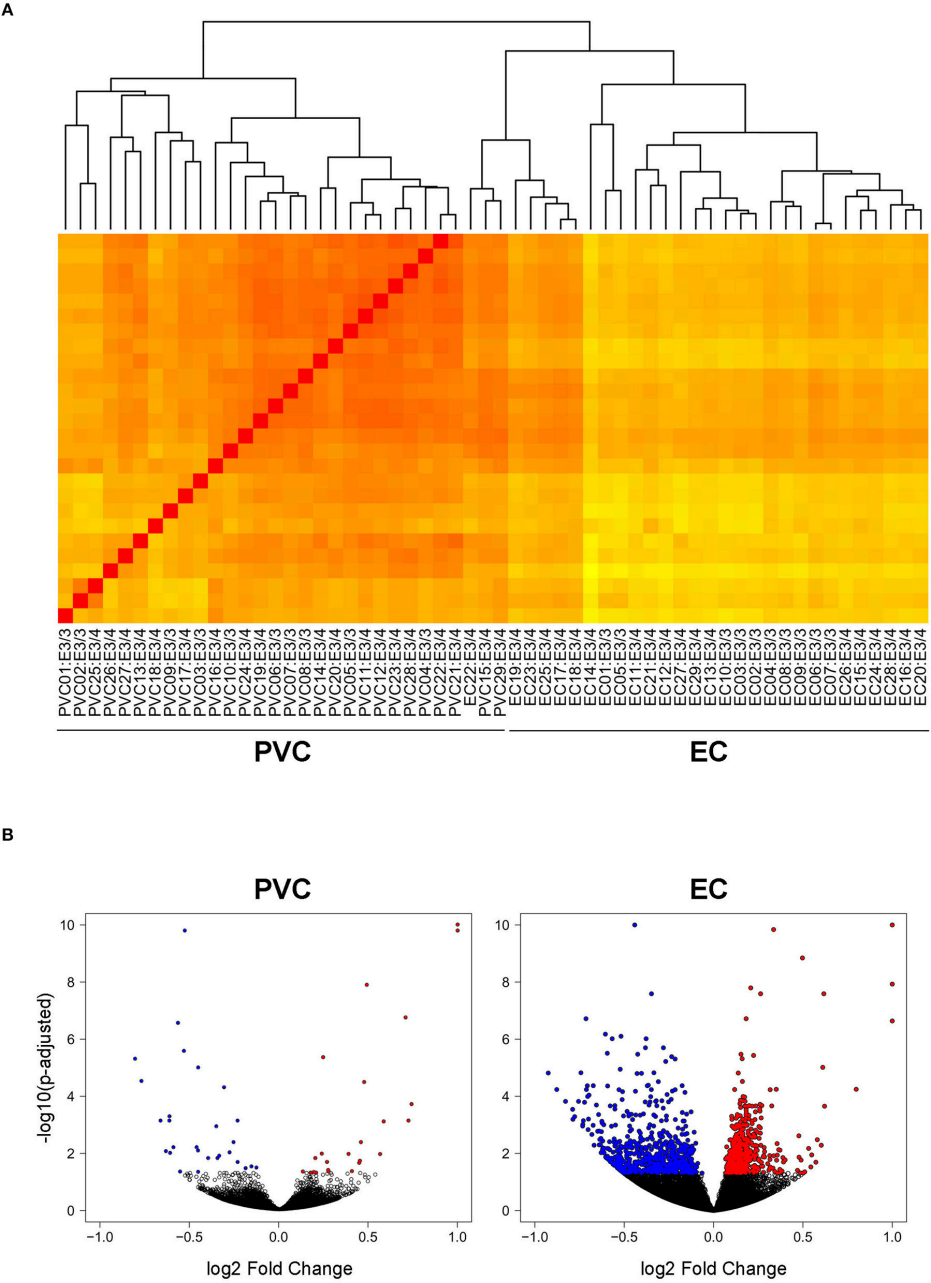
Transcriptomics analysis was performed on RNA extracted from the EC and PVC of aged *APOE3/4* vs. *APOE3/3* mice. RNA-sequencing was performed to analyze the effects of differential *APOE* isoform expression on RNA levels in the PVC and EC of aged *APOE* mice *APOE3/4* (19 *APOE3/4* males vs. 10 *APOE3/3* males). **(A)** A heat map visualizing the Euclidean distance between samples, with darker red colors indicating increased similarity. **(B)** Volcano plots visualizing the fold change and adjusted *p*-values of each gene that was differentially expressed in the PVC and EC of *APOE3/4* vs. *APOE3/3* mice.

In order to investigate the effect of *APOE4* expression in an AD-vulnerable vs. an AD-resistant brain region, RNA-sequencing was performed on RNA extracted from the EC and PVC of 14–15 month-old *APOE* targeted replacement mice expressing human *APOE3/4* (19 males) vs. *APOE3/3* (10 males). As shown in Figure 1A, the samples clustered independently based on brain region, but not based on genotype. This is likely due to the relatively small number of genes that were differentially expressed between *APOE3/4* and *APOE3/3* mice in each region versus the total number of genes analyzed. Using an FDR-corrected *p*-value of less than 0.05, we identified 1292 genes in the EC and 55 genes in the PVC that were differentially expressed between *APOE3/4* and *APOE3/3* (Tables S1, S2), out of a total of 31,246 genes analyzed. In the EC, 683 genes were decreased and 609 genes were increased in *APOE3/4* mice as compared to *APOE3/3* mice, while in the PVC, 28 genes were decreased and 27 genes were increased in APOE3/4 mice as compared to APOE3/3 mice (Figure 1B).

### Genes related to endosomal–lysosomal system function are upregulated in the EC of aged *APOE3/4* vs. *APOE3/3* mice

**Figure 2:**
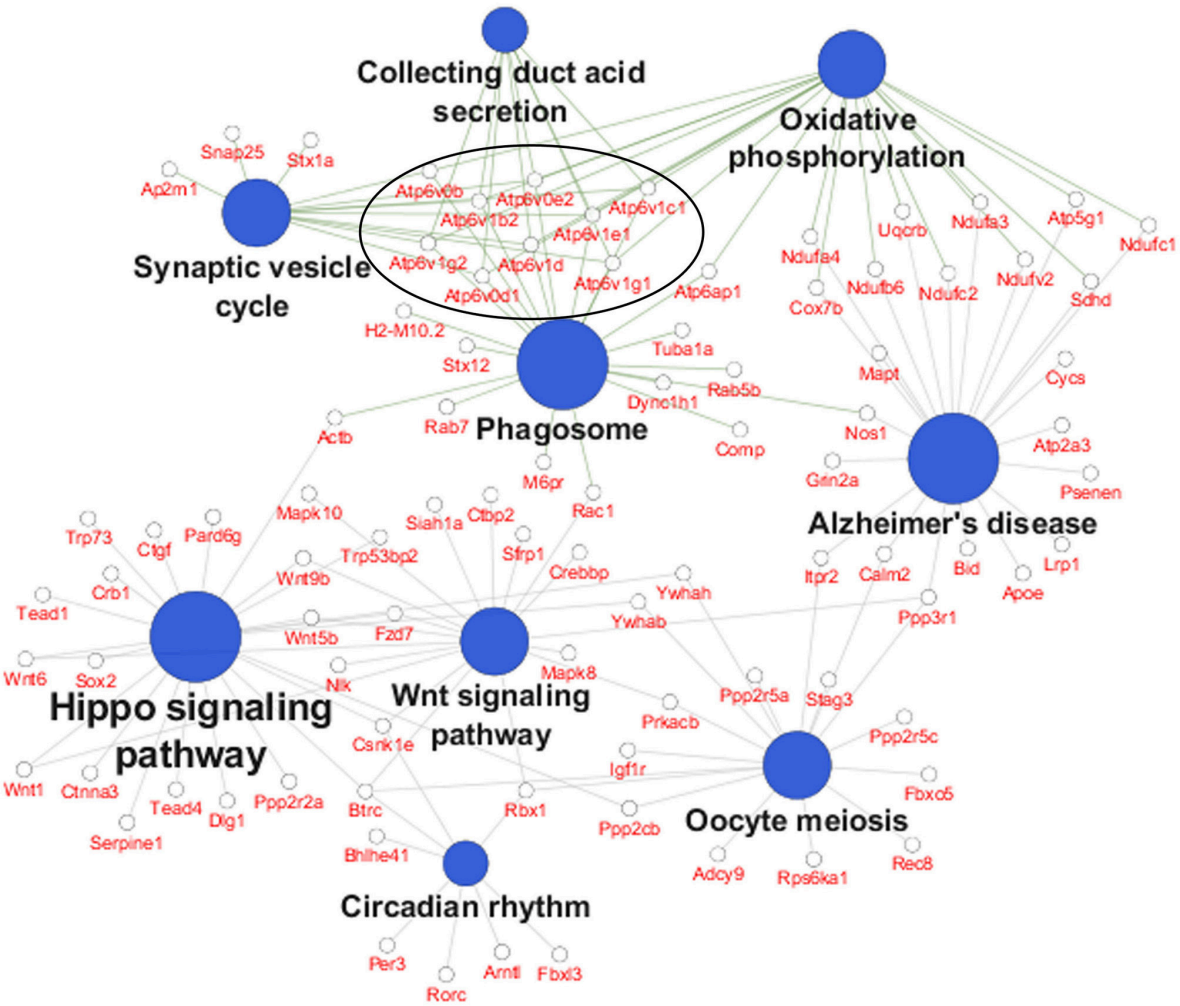
Pathway analysis reveals numerous pathways affected by differential *APOE* isoform expression. A pathway analysis was performed on the differentially expressed EC genes using Cytoscape's ClueGo application. Shown here are the significantly enriched KEGG pathways observed in the EC of *APOE3/4* vs. *APOE3/3* mice, with the 9 differentially expressed V-type ATPase genes circled. (Further details can be found in Table 1).

In order to identify specific cellular pathways that are affected by differential *APOE* isoform expression in the EC, we performed KEGG pathway analysis on the differentially expressed genes from the EC of *APOE3/4* vs. *APOE3/3* mice using Cytoscape's ClueGo application. This analysis identified 9 KEGG pathways that were enriched with genes from the EC of *APOE3/4* vs. *APOE3/3* mice (Figure 2 and Table 1). An informal analysis of these results revealed that there were four major pathway groups affected by the *APOE3/4* genotype in the EC: vesicle function (synaptic vesicle cycle, collecting duct acid secretion and phagosome pathways), developmental genes (Hippo and Wnt signaling pathways), oxidative phosphorylation (oxidative phosphorylation pathway) and AD (Alzheimer's disease pathway).

**Table 1:** Enriched KEGG Pathways in the EC of *APOE3/4* vs. *APOE3/3* mice.

**Gene ontology term**	**KEGG ID**	**Number of genes**	**Percent matched**	***p*-value**	**FDR**
Hippo signaling pathway	4390	23	14.7	2.64E-05	0.004
Synaptic vesicle cycle	4721	12	19.4	1.77E-04	0.006
Oxidative phosphorylation	190	20	14.2	1.52E-04	0.007
Collecting duct acid secretion	4966	8	29.6	9.52E-05	0.007
Circadian rhythm	4710	8	25.8	2.76E-04	0.008
Oocyte meiosis	4114	17	14.7	3.18E-04	0.008
Alzheimer's disease	5010	22	12.3	0.001	0.017
Phagosome	4145	21	12.1	0.001	0.024
Wnt signaling pathway	4310	18	12.6	0.002	0.025

*Shown are all KEGG pathways enriched in the APOE3/4 vs. APOE3/3 EC that possessed a false discovery rate (FDR) of less than 0.05, with additional information on the number of genes that were found in each pathway and the percentage of the total genes that were matched for each pathway*.

**Figure 3:**
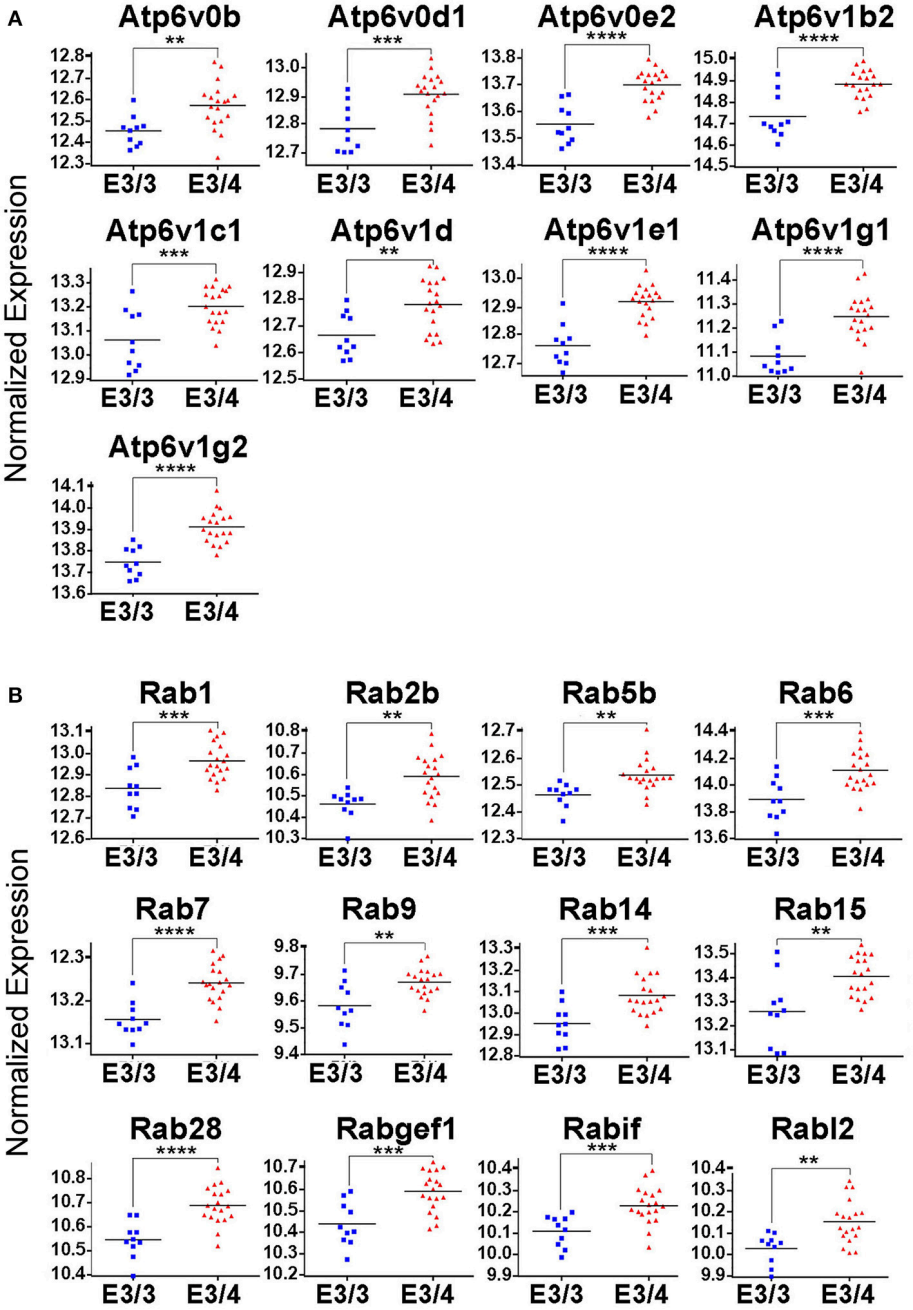
Genes related to endosomal–lysosomal system function are upregulated in the EC of *APOE3/4* mice. Our RNA-Seq analysis of EC genes from aged *APOE3/4* vs. *APOE3/3* mice revealed an enrichment of upregulated genes related to endosomal–lysosomal system function. **(A)** Differentially regulated V-type ATPases in the EC of *APOE3/4* vs. *APOE3/3* mice. **(B)** Differentially regulated Rab GTPases in the EC of *APOE3/4* vs. *APOE3/3* mice. (^**^p < 0.01; ^***^p < 0.001; ^****^p < 0.0001).

While all of these KEGG pathway groups are intriguing, we focused on the vesicle function group. As highlighted in Figure 2, an important contributor to the difference in the vesicle function group was an increase in the expression of 9 Vacuolar H^+^-ATPases (V-ATPases) in the EC of *APOE3/4* mice (Figure 3A). V-ATPases are vital for the acidification and function of a wide range of vesicular compartments, including phagosomes in macrophages and microglia, synaptic vesicles in neurons, and endosomes, lysosomes and autophagosomes in numerous cell types (Marshansky et al., 2014; Cotter et al., 2015). In order to further explore this pathway enrichment, we also performed a DAVID analysis for Biological Process (BP), Cellular Component (CC) and Molecular Function (MF) Gene Ontology (GO) terms (Table 2), and we found that differentially expressed EC genes were enriched in “endosome,” “lysosomal membrane” and “extracellular exosome” GO terms, in addition to numerous other vesicle and transport-related GO terms. For this reason, we chose to direct our attention to the effect of differential *APOE* isoform expression on the endosomal–lysosomal system.

**Table 2:** Enriched Gene Ontology Terms in the EC of *APOE3/4* vs. *APOE3/3* mice.

**Gene ontology term**	**Gene ontology database**	**Number of genes**	**Percent matched**	***p*-value**	**FDR**
Protein binding	GOTERM_MF_DIRECT	315	28.2	2E-16	2.4E-13
Cytoplasm	GOTERM_CC_DIRECT	430	38.5	1.8E-12	1.1E-09
Cytosol	GOTERM_CC_DIRECT	150	13.4	6.6E-11	2.1E-08
Membrane	GOTERM_CC_DIRECT	439	39.3	1.7E-10	3.6E-08
**Extracellular exosome**	GOTERM_CC_DIRECT	198	17.7	1.9E-09	3E-07
Mitochondrion	GOTERM_CC_DIRECT	134	12	9.5E-08	0.000012
Cytoplasmic vesicle membrane	GOTERM_CC_DIRECT	22	2	3.8E-07	0.00004
Cell junction	GOTERM_CC_DIRECT	67	6	0.0000008	0.000071
Myelin sheath	GOTERM_CC_DIRECT	27	2.4	0.0000029	0.00022
Transport	GOTERM_BP_DIRECT	141	12.6	0.0000003	0.0011
Focal adhesion	GOTERM_CC_DIRECT	40	3.6	0.000024	0.0017
Axon	GOTERM_CC_DIRECT	38	3.4	0.000037	0.0021
**Lysosomal membrane**	GOTERM_CC_DIRECT	28	2.5	0.000036	0.0023
Neuron projection	GOTERM_CC_DIRECT	40	3.6	0.00012	0.0052
Golgi membrane	GOTERM_CC_DIRECT	39	3.5	0.0001	0.0053
SNARE complex	GOTERM_CC_DIRECT	11	1	0.00011	0.0055
**Endosome**	GOTERM_CC_DIRECT	48	4.3	0.00014	0.0058
Cytoskeleton	GOTERM_CC_DIRECT	82	7.3	0.00027	0.01
Nucleoplasm	GOTERM_CC_DIRECT	129	11.6	0.00032	0.012
Perinuclear region of cytoplasm	GOTERM_CC_DIRECT	56	5	0.00034	0.012
Cell surface	GOTERM_CC_DIRECT	51	4.6	0.00061	0.02
Vesicle	GOTERM_CC_DIRECT	19	1.7	0.0012	0.038
Melanosome	GOTERM_CC_DIRECT	14	1.3	0.0014	0.038
Synaptic vesicle	GOTERM_CC_DIRECT	16	1.4	0.0014	0.04
Rhythmic process	GOTERM_BP_DIRECT	20	1.8	0.000025	0.043
Postsynaptic density	GOTERM_CC_DIRECT	24	2.2	0.0017	0.044
Centrosome	GOTERM_CC_DIRECT	37	3.3	0.0017	0.046

*All GO terms enriched in the APOE3/4 vs. APOE3/3 EC that possessed a false discovery rate (FDR) of less than 0.05, with additional information on the GO database they were found in, the number of genes that were found from each GO term and the percent of genes that were matched for each GO term (GO terms directly related to the endosomal–lysosomal system are in bold)*.

In addition to the increased expression of V-ATPases, further mining of the RNA-Seq data revealed 12 different Rab GTPases and associated genes (Figure 3B), including Rab5b and Rab7, which are vital for the formation and function of early and late endosomes, respectively, as well as several other important regulators of endosomal–lysosomal function, including Atp6ap1, Atp6ap2, Snx3, Snx15, Vps4a, Vps24, Vps29, Vta1, and Mp6r. Intriguingly, all of these genes were found to be upregulated in the *APOE3/4* EC, which we hypothesize might represent a compensatory effect in response to a broad dysregulation of the endosomal–lysosomal system in the EC of these *APOE3/4* mice. Importantly, none of these genes were shown to be differentially expressed in the PVC, suggesting that *APOE4*-mediated endosomal–lysosomal pathology may be occurring predominantly in AD-vulnerable brain regions such as the EC.

### The morphology of endosomal–lysosomal compartments is altered in the brains of aged *APOE4/4* mice

**Figure 4:**
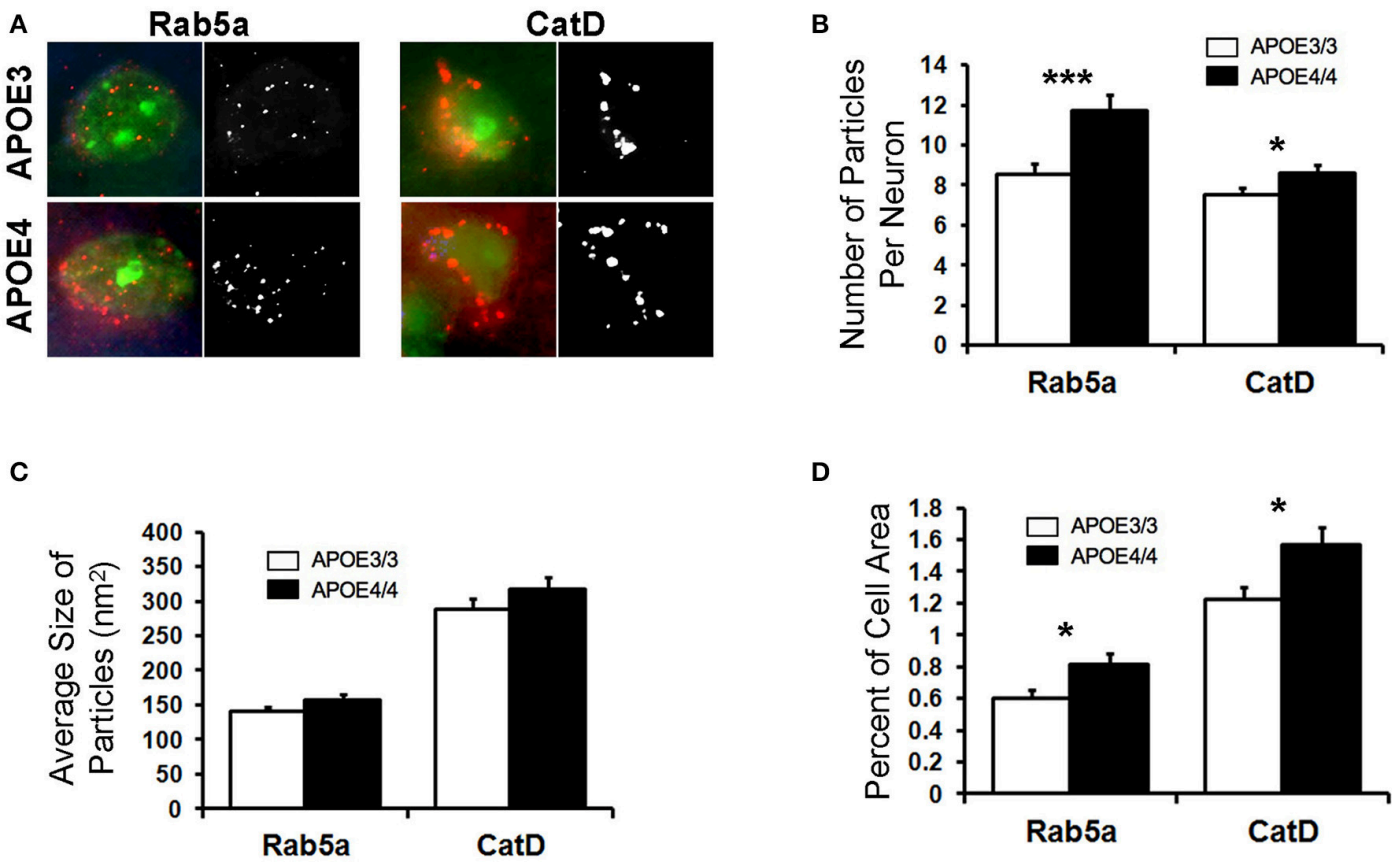
The number and area fraction of Rab5a- and CatD-immunoreactive compartments are increased in the EC of aged *APOE4/4* vs. *APOE3/3* mice. Rab5a-immunoreactive early endosomes and CatD-immunoreactive lysosomes were quantified in the entorhinal cortex of 18 month-old *APOE3/3* and *APOE4/4* mice. **(A)** Representative images showing Rab5a and CatD immunolabeling (red), co-stained with Nissl (green). Black and white images were generated in ImageJ and used for quantification. **(B–D)** Quantification of average Rab5a- and CatD-positive compartment number **(B)**, size **(C)**, and area fraction **(D)** in *APOE4/4* vs. *APOE3/3* mice. (^*^p < 0.05; ^***^p < 0.001).

Abnormalities in the endosomal–lysosomal system are prominent in AD (see review by Nixon, 2017). Enlarged early endosomes are among the first cellular pathological features observed in AD pathogenesis (Cataldo et al., 2000, 2004; Nixon et al., 2000; Nixon, 2005), even prior to amyloid deposits in a given brain region, and AD-related enlarged early endosomes occur to a greater degree in the brains of *APOE4* carriers (Cataldo et al., 2000). Building upon our transcriptomics findings and to determine whether endosomes are similarly affected by *APOE4* expression in the absence of overt AD pathology, we investigated the endosomal–lysosomal system in the brains of 18-month-old *APOE4/4* vs. *APOE3/3* targeted replacement mice, as has been done in human tissue (Cataldo et al., 2000). 18-month-old *APOE4/4* vs. *APOE3/3* mice were chosen in order to gain a more robust picture of the endosomal–lysosomal changes observed in the transcriptomics results. Focusing initially on the EC of these mice, we quantified Rab5a immunolabeling of early endosomes in pyramidal neurons (Figure 4), as we have done previously on other AD-related mouse models (Choi et al., 2013; Jiang et al., 2016). In *APOE4/4* mice, we observed an increase in the mean number (8.5 ± 0.5 *APOE3/3*, 11.7 ± 0.8 *APOE4/4* particles/cell; *p* = 0.0007; Figure 4B) and area fraction (0.6 ± 0.05% *APOE3/3*, 0.8 ± 0.07% *APOE4/4* of cell area; *p* = 0.02; Figure 4D) of Rab5a-positive neuronal early endosomes when compared with age-matched *APOE3* mice. No significant differences were seen in average endosomal size between the genotypes (140 ± 7.0 *APOE3/3*, 157 ± 8.0 *APOE4/4* in nm2; *p* = 0.12; Figure 4C).

To analyze the effect of *APOE4* genotype on distal endosomal–lysosomal pathway compartments in neurons, we performed immunolabeling for cathepsin D (CatD), an acid protease that is localized primarily to lysosomes (Figure 4). As with the Rab5a-positive early endosomes, we saw an increase in the mean number (7.5 ± 0.3 *APOE3/3*, 8.6 ± 0.4 *APOE4/4*; *p* = 0.04; Figure 4B) and area fraction (1.2 ± 0.8 *APOE3/3*, 1.6 ± 0.11 *APOE4/4*; *p* = 0.02; Figure 4D) of CatD-positive compartments in the EC of *APOE4/4* mice, as compared to *APOE3/3* controls. Average size of the CatD-positive compartments was not changed between genotypes (288.5 ± 15.8 *APOE3/3*, 317.1 ± 22.3 *APOE4/4*; *p* = 0.22; Figure 4C).

**Figure 5:**
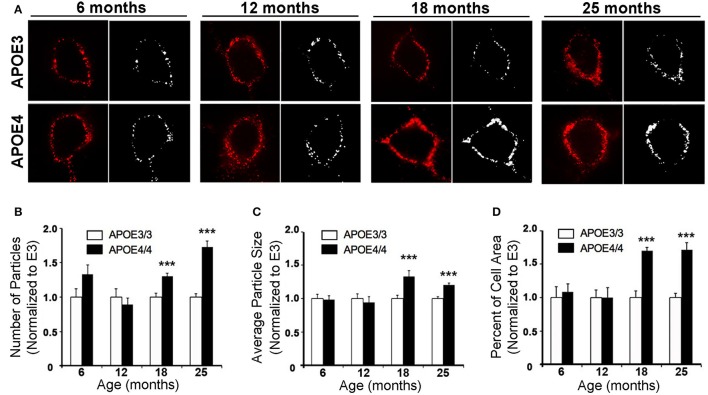
The number, size and area fraction of Rab5a-immunoreactive early endosomes are increased in the cingulate cortex of *APOE4/4* vs. *APOE3/3* mice with age. Rab5a-immunoreactive early endosomes were quantified in the cingulate cortex of 6, 12, 18, and 25 month-old *APOE3/3* and *APOE4/4* mice. **(A)** Representative images showing Rab5a immunolabeling (red). Black and white images were generated in ImageJ and used for quantification. **(B–D)** Quantification of average Rab5a-positive early endosome number **(B)**, size **(C)**, and area fraction **(D)** in *APOE4/4* vs. *APOE3/3* mice. (^***^p < 0.001; findings are normalized to the mean *APOE3/3* value at each age in order to compare between age groups).

In order to further characterize the effect of *APOE4* on early endosomes, we performed a larger study of Rab5a-positive early endosomes in the cingulate cortex of 6, 12, 18, and 25 month old *APOE4/4* vs. *APOE3/3* mice (Figure 5). The cingulate cortex was chosen because, like the EC, it is vulnerable to AD pathology (Leech and Sharp, 2014) and to *APOE4*-specific deficits (Valla et al., 2010). Additionally, we previously characterized early endosomal alterations in this region in amyloid precursor protein transgenic mice (Choi et al., 2013) and, as it is larger than the EC, we anticipated that characterizing early endosomes in this region would facilitate our aging analysis. While no differences were seen in early endosome size, number or area fraction at 6 or 12 months of age, an increase in mean early endosome number (an increase of 30.5 ± 4.0%; Figure 5B), size (33.5 ± 8.0%; Figure 5C), and area fraction (70.1 ± 4.8%; Figure 5D) was seen in the 18 month-old *APOE4/4* mice as compared to *APOE3/3* controls, a difference that persisted in the 25 month-old animals (endosome number: 73.3 ± 8.4%; size: 20.7 ± 2.7%; area fraction: 71.8 ± 10.4%). This magnitude of early endosome change is similar to that which was reported in Down syndrome mouse models (Cataldo et al., 2003), and is similar to the APOE4-dependent early endosomal alteration seen in human neurons in the prefrontal cortex in early-stage AD (Cataldo et al., 2000).

## Discussion

Carriers of the ε4 allele of *APOE* are at a significantly increased risk of developing late-onset AD (Farrer et al., 1997). However, the exact cause of *APOE4*'s association with AD is not fully understood. One potential mediator of *APOE4*'s pathogenicity in AD may be disruption of the endosomal–lysosomal system, a process that is vital to cellular function and viability. Intriguingly, enlarged early endosomes are observed early in the pathogenesis of AD (Cataldo et al., 2000, 2004; Nixon et al., 2000; Nixon, 2005), and are present to a greater degree in the brains of *APOE4* carriers (Cataldo et al., 2000). However, it is not clear if incipient AD pathology may play a role in the enlarged early endosome phenotype in *APOE4* carriers. Interestingly, familial AD (FAD) caused by presenilin mutations does not exhibit the same abnormal endosomal phenotype (Cataldo et al., 2000), nor is presenilin mutation-induced FAD influenced by *APOE* genotype (Van Broeckhoven, 1995; Houlden et al., 1998), suggesting that amyloid pathology does not directly cause this early endosomal enlargement. While several studies have observed a deleterious effect of *APOE4* on the fidelity of the endosomal–lysosomal system *in vitro*, the majority of these studies have been performed using immortalized cell lines with either exogenously added recombinant apoE protein or *APOE* overexpression systems (DeKroon and Armati, 2001; Ji et al., 2002; Yamauchi et al., 2002; Heeren et al., 2004; Ye et al., 2005; Rellin et al., 2008; Chen et al., 2010; Li et al., 2012). Therefore, we sought to determine if *APOE4* isoform expression impairs the endosomal–lysosomal pathway in an endogenous, *in vivo* setting that is free of AD pathology.

Using a well-characterized mouse model of human *APOE* isoform expression that does not develop amyloid plaques or neurofibrillary tangles (Sullivan et al., 1997, 2004), we were able to demonstrate that *APOE4* expression directly causes endosomal–lysosomal system dysregulation using both transcriptomics and immunohistochemistry. In our RNA-sequencing analysis, we discovered an *APOE4*-dependent upregulation of a wide array of endosome and lysosome-related genes, including 9 V-type ATPases and 12 Rab GTPases, in the EC of aged *APOE3/4* mice (Figure 3), which is suggestive of general endosomal–lysosomal pathway dysfunction. Furthermore, two of the Rab GTPase genes that we found to be upregulated in the transcriptomics analysis, the early endosome effector Rab5 and the late endosome effector Rab7, have also been found to be consistently upregulated in human cases of AD in selectively vulnerable neuronal populations (Ginsberg et al., 2010a,b, 2011). Additionally, gene expression of CatD is also upregulated in AD pyramidal neurons (Cataldo et al., 1995). Endosomal–lysosomal system abnormalities were confirmed through immunohistochemistry, which showed an increase in the size and area fraction of Rab5a-containing early endosomes and CatD-containing lysosomes in the EC of aged *APOE4/4* mice (Figure 4). An age-dependent increase in the number, size and area fraction of Rab5a-containing early endosomes in the cingulate cortex of 18- and 25-month old (but not 6- and 12-month old) *APOE4/4* mice (Figure 5) was also seen. Together, these findings demonstrate that *APOE4* expression causes an age-dependent alteration in the endosomal–lysosomal system in the brain that is independent of overt AD pathology. Given the importance of the endosomal–lysosomal pathway in both the production of Aβ (see review by Thinakaran and Koo, 2008) and the clearance of pathogenic proteins (see review by Nixon, 2017), we believe that *APOE4*-associated endosomal–lysosomal system dysregulation may be an early instigator of AD pathogenesis among *APOE4* carriers.

In addition to our observation of an enrichment in differentially expressed endosomal–lysosomal genes, our transcriptomics analysis revealed many other intriguing genes that were found to be differentially expressed in the EC of aged *APOE3/4* vs. *APOE3/3* mice, which we hope will spur additional research on the possible effects of these genes in *APOE4*-associated AD pathogenesis. The observation that differential *APOE* isoform expression affects two major developmental pathways (Hippo signaling and Wnt signaling; Figure 2) is of potential interest for *APOE* biology in general, since *APOE4* has been shown to alter brain development, with infants carrying the *APOE4* allele possessing region-specific differences in their white matter myelin water fractions (MWF) and gray matter volumes (GMV) compared to non-carriers (McDonald and Krainc, 2014) and juveniles carrying the *APOE4* allele exhibiting reduced EC volumes compared to non-carriers (Shaw et al., 2007). Furthermore, *APOE4* has been shown to inhibit Wnt signaling *in vitro* (Caruso et al., 2006).

Another intriguing finding was that several genes involved in the mitochondrial oxidative phosphorylation process (*Atp5g1, Atp6ap1, Cox7b, Ndufa3, Ndufa4, Ndufb6, Ndufc2, Ndufv2*, and *Sdhd*) are upregulated by *APOE4*. Although other studies have shown reduced glucose utilization in various brain regions of *APOE4* carriers (Reiman et al., 1996, 2001, 2004), these results suggest that *APOE4* may increase glucose-driven oxidative phosphorylation in the EC. In addition, we found a specific enrichment in genes that have previously been shown to be related to Alzheimer's disease pathogenesis, including *Mapt* and *Psenen*. These results warrant further investigation to determine the roles of these important genes in *APOE4*-associated AD susceptibility.

In total, these findings inform on *APOE* neurobiology and how it may act to increase AD pathogenesis, most notably through a dysregulation of the endosomal–lysosomal system. In addition, we anticipate that the transcriptomics results presented here will facilitate additional research into the link between *APOE4* and AD.

## Author contributions

This study was designed and managed by TN, KP, PM, and KD. Animal care and breeding was performed by HF. RNA extraction, RNA-Sequencing and data processing was performed by TN and AD in the laboratory of MC. Pathway analysis was performed by TN in the laboratory of KD. Immunohistochemistry was performed by KP in the laboratories of PM and EL Quantification of endosomal and lysosomal compartments was performed by KP, AA, JuA, JaA, and TN. Manuscript preparation was performed by TN, KP, and PM with input from EL, MC, and KD.

### Conflict of interest statement

The authors declare that the research was conducted in the absence of any commercial or financial relationships that could be construed as a potential conflict of interest.

## Supplementary material

Table S1 Differentially expressed genes from the EC of *APOE3/4* vs. *APOE3/3* mice.

Table S2 Differentially expressed genes from the PVC of *APOE3/4* vs. *APOE3/3* mice.
