# Supplementary material for: The Endosomal–Lysosomal Pathway Is Dysregulated by APOE4 Expression in Vivo
Source: Front Neurosci. 2017 Dec 12;11:702. doi: 10.3389/fnins.2017.00702 (PMC5733017; doi:10.3389/fnins.2017.00702)
Supplement: Table S1 — Differentially expressed genes from the EC of APOE3/4 vs. APOE3/3 mice. [file Table1.PDF]

Supplementary Material

The Endosomal-Lysosomal Pathway is Dysregulated by *APOE4* Expression *In Vivo*

Tal Nuriel, Katherine Y. Peng, Archana Ashok, Allissa A. Dillman, Helen Y. Figueroa, Justin Apuzzo, Jayanth Ambat, Efrat Levy, Mark R. Cookson, Paul M. Mathews, and Karen E. Duff

[*]These two authors contributed equally to this work and are co-first authors.

Correspondance: Paul Matthews, Paul.Mathews@nki.rfmh.org; Karen Duff, ked2115@columbia.edu

Supplementary Tables

Table S1. Differentially expressed genes from the EC of *APOE3/4* vs. *APOE3/3* mice

Gene Name	log2 FoldChange	p-value	FDR
**Serpina3m**	3.97	1.86E-166	3.41E-162
**Serpina3n**	1.95	4.65E-147	4.26E-143
**Oscar**	2.20	1.09E-60	6.67E-57
**Rdh13**	-0.44	1.37E-24	6.29E-21
**Myadm**	0.34	3.96E-14	1.45E-10
**Thnsl1**	0.50	4.69E-13	1.43E-09
**Gm12762**	1.14	4.52E-12	1.18E-08
**Mboat7**	0.21	6.98E-12	1.60E-08
**Ica1**	-0.35	1.55E-11	2.57E-08
**Syngr3**	0.26	1.38E-11	2.57E-08
**Tmc4**	0.62	1.42E-11	2.57E-08
**Asprv1**	-0.71	1.36E-10	1.92E-07
**Fbxl2**	0.18	1.35E-10	1.92E-07
**Gm1082**	1.04	1.76E-10	2.30E-07
**Gm1078**	-0.61	5.46E-10	6.67E-07
**Wdfy1**	-0.52	6.90E-10	7.90E-07
**Srcap**	-0.38	9.06E-10	9.63E-07
**Zbtb20**	-0.57	9.46E-10	9.63E-07
**Dpysl2**	-0.38	2.18E-09	2.00E-06
**Pon2**	-0.28	2.13E-09	2.00E-06
**Gm15494**	-0.59	3.57E-09	3.11E-06
**Ccdc32**	0.15	4.23E-09	3.37E-06
**Mll2**	-0.42	4.17E-09	3.37E-06
**Fam103a1**	0.22	4.86E-09	3.71E-06
**Pkd1**	-0.23	5.58E-09	4.09E-06
**Atp6v1e1**	0.16	6.86E-09	4.83E-06
**Zfp652**	-0.22	7.28E-09	4.94E-06
**Dchs1**	-0.27	9.22E-09	6.03E-06
**Gm4723**	0.61	1.53E-08	9.68E-06
**1520402A15Rik**	-0.52	1.85E-08	1.13E-05
**Cml5**	-0.74	2.63E-08	1.50E-05
**Nfia**	-0.33	2.59E-08	1.50E-05
**Atp6v0e2**	0.14	2.85E-08	1.53E-05

**Trcg1**	-0.92	2.79E-08	1.53E-05
**AC154449.2**	-0.40	3.01E-08	1.57E-05
**Zfhx3**	-0.43	3.13E-08	1.59E-05
**Atp6v1g2**	0.16	6.11E-08	3.02E-05
**Mios**	-0.31	6.36E-08	3.07E-05
**Lrrc58**	-0.37	8.70E-08	4.09E-05
**Ppp6r2**	-0.18	9.50E-08	4.24E-05
**Trim72**	-0.67	9.33E-08	4.24E-05
**AC132303.1**	-0.37	1.00E-07	4.27E-05
**Cftr**	-0.71	9.84E-08	4.27E-05
**Cybrd1**	-0.59	1.05E-07	4.35E-05
**Ptprh**	0.80	1.39E-07	5.67E-05
**Cdh10**	0.35	1.43E-07	5.69E-05
**AC102195.1**	-0.88	1.51E-07	5.78E-05
**Mapt**	0.12	1.51E-07	5.78E-05
**Gm10931**	-0.71	1.55E-07	5.81E-05
**Gm3435**	-0.35	1.59E-07	5.82E-05
**BX537302.1**	-0.53	1.67E-07	5.87E-05
**Itpr2**	-0.21	1.66E-07	5.87E-05
**Rgs4**	0.32	1.70E-07	5.87E-05
**Tgfa**	-0.23	1.93E-07	6.54E-05
**Sbsn**	-0.44	2.00E-07	6.66E-05
**Gm11944**	-0.51	2.10E-07	6.87E-05
**Prdm11**	-0.16	2.27E-07	7.30E-05
**Tmem170b**	-0.26	2.74E-07	8.65E-05
**Plin4**	-0.70	2.93E-07	9.09E-05
**Ahsa1**	0.17	3.39E-07	0.000102
**Slco2a1**	-0.70	3.37E-07	0.000102
**Ksr2**	-0.35	3.48E-07	0.000103
**2010107E04Rik**	0.17	3.65E-07	0.000106
**Pan3**	-0.24	4.46E-07	0.000128
**RP24-161J8.2**	-0.51	4.62E-07	0.00013
**Nars**	0.15	4.94E-07	0.000137
**AC122417.1**	-0.32	5.40E-07	0.000143
**Spnb1**	-0.29	5.24E-07	0.000143
**Srp19**	0.16	5.34E-07	0.000143
**1500031L02Rik**	0.18	6.00E-07	0.000151
**Dctd**	-0.32	5.81E-07	0.000151
**Gm12116**	-0.76	5.94E-07	0.000151
**Gm16997**	-0.83	5.93E-07	0.000151

**Adcy9**	-0.25	6.33E-07	0.000157
**Acacb**	-0.38	6.49E-07	0.000158
**Igsf3**	-0.36	6.54E-07	0.000158
**Hif3a**	-0.72	6.81E-07	0.000162
**Syt11**	0.15	7.71E-07	0.000181
**Vopp1**	0.26	8.24E-07	0.000191
**Fmo2**	-0.66	8.89E-07	0.000202
**Ranbp17**	-0.36	8.94E-07	0.000202
**Srp54b**	0.27	9.45E-07	0.000211
**0610038L08Rik**	-0.55	9.59E-07	0.000212
**Mrpl36**	0.20	9.74E-07	0.000212
**Aff1**	-0.32	9.95E-07	0.000214
**Rdh9**	-0.58	1.03E-06	0.000217
**Synj2**	0.33	1.03E-06	0.000217
**Stmn1**	0.24	1.05E-06	0.000218
**Hbxip**	0.19	1.08E-06	0.000221
**Zc3h7b**	0.22	1.09E-06	0.000221
**Anxa4**	0.62	1.12E-06	0.000222
**Ndufb6**	0.17	1.13E-06	0.000222
**Yeats4**	0.18	1.12E-06	0.000222
**Ngrn**	0.15	1.26E-06	0.000246
**Bid**	-0.28	1.35E-06	0.00026
**Ncald**	0.26	1.44E-06	0.000275
**Flnc**	-0.55	1.48E-06	0.00028
**CT868690.1**	-0.79	1.53E-06	0.000286
**2410004B18Rik**	0.18	1.61E-06	0.000298
**Atf4**	0.26	1.80E-06	0.00033
**Icosl**	-0.48	1.86E-06	0.000338
**Tmem60**	0.23	1.97E-06	0.00035
**Zfp334**	-0.13	1.97E-06	0.00035
**Txndc12**	0.18	2.08E-06	0.000367
**Dync1h1**	-0.17	2.11E-06	0.000369
**Sdc4**	-0.39	2.39E-06	0.000412
**Scube2**	-0.47	2.45E-06	0.000419
**Agl**	-0.15	2.48E-06	0.00042
**Ahnak**	-0.46	2.53E-06	0.000425
**Pkp2**	-0.64	2.64E-06	0.000435
**Tmem85**	0.13	2.62E-06	0.000435
**Snrpd3**	0.18	2.69E-06	0.00044
**Celsr1**	-0.33	2.77E-06	0.00045

**Ahnak2**	-0.44	2.80E-06	0.00045
**Gm13375**	-0.41	2.87E-06	0.000458
**Zfand6**	0.20	2.96E-06	0.000467
**Selk**	0.15	3.07E-06	0.000481
**Gpnmb**	-0.47	3.15E-06	0.000487
**Gpr182**	-0.50	3.17E-06	0.000487
**Zhx3**	-0.26	3.19E-06	0.000487
**Rab7**	0.10	3.32E-06	0.000503
**Zbtb44**	-0.32	3.38E-06	0.000507
**RP23-388P16.8**	-0.78	3.46E-06	0.000515
**Ncbp2**	0.13	3.54E-06	0.000523
**Slc25a22**	0.18	3.88E-06	0.000568
**Cobl**	0.27	3.93E-06	0.000572
**Cd99l2**	0.13	4.05E-06	0.000585
**Ndfip1**	0.20	4.44E-06	0.000626
**Slc1a4**	0.16	4.39E-06	0.000626
**Trp73**	-0.70	4.42E-06	0.000626
**Ankrd7**	-0.79	4.57E-06	0.00064
**Prdx2**	0.10	4.62E-06	0.000641
**Agxt2l2**	-0.21	4.78E-06	0.000651
**Ly6e**	0.23	4.76E-06	0.000651
**Pm20d1**	-0.45	4.80E-06	0.000651
**Dhdh**	-0.26	5.03E-06	0.000677
**Gm15594**	-0.73	5.33E-06	0.000712
**Ppp1r16b**	-0.29	5.42E-06	0.000719
**Med1**	-0.17	5.67E-06	0.000733
**Pak1**	0.18	5.68E-06	0.000733
**Uqcrb**	0.22	5.68E-06	0.000733
**Wnk1**	-0.16	5.64E-06	0.000733
**Gm10151**	-0.62	5.85E-06	0.000749
**Isca2**	0.15	6.19E-06	0.000787
**4631405J19Rik**	-0.62	6.80E-06	0.000859
**Nlk**	0.22	7.22E-06	0.000906
**Gm10595**	-0.73	7.72E-06	0.000962
**Atp6v1b2**	0.14	8.34E-06	0.000991
**Atp6v1g1**	0.17	8.39E-06	0.000991
**Cldnd1**	0.13	8.13E-06	0.000991
**Cyb5**	0.19	8.32E-06	0.000991
**Dnajb6**	0.14	8.05E-06	0.000991
**Fxyd4**	-0.61	8.41E-06	0.000991

**Lonrf3**	-0.36	8.50E-06	0.000991
**Nicn1**	0.14	8.40E-06	0.000991
**Stx12**	0.15	8.51E-06	0.000991
**Tspyl4**	0.18	8.55E-06	0.000991
**Ube2e1**	0.13	8.51E-06	0.000991
**BC046401**	-0.55	8.84E-06	0.001018
**Rab28**	0.14	8.90E-06	0.001019
**Map1lc3b**	0.19	9.11E-06	0.001037
**Lefty1**	-0.67	9.18E-06	0.001038
**Ube2b**	0.18	9.52E-06	0.00107
**Lama1**	-0.47	9.77E-06	0.001092
**Sntb2**	-0.33	9.87E-06	0.001096
**Gm15082**	-0.73	9.96E-06	0.001099
**Cd2bp2**	0.10	1.00E-05	0.0011
**Mtap1a**	-0.16	1.02E-05	0.001114
**Slc2a9**	-0.57	1.03E-05	0.001118
**Rexo2**	0.14	1.06E-05	0.001143
**Atcay**	0.15	1.07E-05	0.001144
**Otud1**	0.25	1.07E-05	0.001144
**Slc25a3**	0.12	1.12E-05	0.001186
**Arl6ip5**	0.13	1.13E-05	0.001193
**Lysmd4**	0.13	1.17E-05	0.001222
**Ywhah**	0.14	1.17E-05	0.001222
**Atp6ap1**	0.11	1.24E-05	0.001274
**Fnbp1l**	0.26	1.24E-05	0.001274
**Stab2**	-0.36	1.24E-05	0.001274
**Fbxl18**	-0.20	1.28E-05	0.001298
**Letmd1**	0.12	1.38E-05	0.001389
**Txnl4a**	0.21	1.38E-05	0.001389
**Lysmd2**	0.17	1.40E-05	0.001405
**Nup98**	-0.17	1.43E-05	0.001423
**Gm12394**	-0.46	1.48E-05	0.001465
**7SK.295**	-0.70	1.49E-05	0.001469
**Atg5**	0.15	1.53E-05	0.001496
**Ndfip2**	0.23	1.58E-05	0.001541
**Elmo3**	-0.28	1.65E-05	0.001601
**Stx8**	0.19	1.71E-05	0.001644
**AC159277.1**	-0.47	1.74E-05	0.001654
**Col6a6**	-0.64	1.74E-05	0.001654
**Trpm3**	-0.42	1.74E-05	0.001654

**SNORD38.1**	-0.65	1.78E-05	0.001676
**Prox1**	-0.63	1.84E-05	0.001728
**2610507B11Rik**	-0.11	1.95E-05	0.001805
**Ndrg4**	0.15	1.93E-05	0.001805
**Rasgef1b**	0.24	1.95E-05	0.001805
**Notch2**	-0.29	2.03E-05	0.001861
**Snrnp27**	0.15	2.02E-05	0.001861
**Mrrf**	0.14	2.05E-05	0.001869
**Hipk2**	-0.26	2.08E-05	0.001882
**Lpp**	-0.35	2.09E-05	0.001883
**Arpp19**	0.26	2.20E-05	0.001957
**Gm15893**	-0.41	2.20E-05	0.001957
**Rbm18**	0.15	2.18E-05	0.001957
**1700021F05Rik**	0.16	2.29E-05	0.00201
**Slc24a4**	-0.30	2.29E-05	0.00201
**Ubr4**	-0.15	2.28E-05	0.00201
**AI413582**	0.19	2.38E-05	0.002073
**Slc39a10**	0.22	2.45E-05	0.00213
**Spcs2**	0.13	2.48E-05	0.002147
**1110054O05Rik**	0.13	2.54E-05	0.002151
**Hsp90b1**	0.20	2.53E-05	0.002151
**Nup214**	-0.16	2.50E-05	0.002151
**Patz1**	-0.18	2.53E-05	0.002151
**Galntl2**	-0.68	2.56E-05	0.002164
**Zfp783**	-0.25	2.73E-05	0.002293
**Got1**	0.16	2.87E-05	0.002403
**Maml2**	-0.30	2.89E-05	0.00241
**Qrfpr**	0.48	2.92E-05	0.002422
**Cd200**	0.23	2.97E-05	0.002448
**Sra1**	0.16	3.00E-05	0.002464
**Cpne4**	0.23	3.05E-05	0.002481
**Serf1**	0.23	3.04E-05	0.002481
**Igsf10**	-0.35	3.15E-05	0.002547
**Ralyl**	0.21	3.17E-05	0.002547
**Tsku**	-0.48	3.17E-05	0.002547
**Foxo3**	-0.24	3.22E-05	0.002577
**Crebbp**	-0.21	3.24E-05	0.002579
**Pnpla7**	-0.28	3.32E-05	0.002622
**Ykt6**	0.12	3.32E-05	0.002622
**B230120H23Rik**	-0.35	3.35E-05	0.002637

**Lrrc29**	-0.41	3.44E-05	0.002682
**Rgs20**	0.23	3.44E-05	0.002682
**Klhl11**	-0.29	3.48E-05	0.002688
**Ptprn2**	0.23	3.47E-05	0.002688
**Cisd2**	0.14	3.57E-05	0.002734
**Pacsin2**	0.22	3.57E-05	0.002734
**Gm15609**	-0.41	3.75E-05	0.002862
**Hnrnph2**	0.17	3.86E-05	0.00292
**Nf1**	-0.21	3.84E-05	0.00292
**Nap1l3**	0.26	3.94E-05	0.002969
**AL662835.2**	-0.63	3.99E-05	0.002981
**Tmem14c**	0.18	3.99E-05	0.002981
**Ube2e2**	0.13	4.12E-05	0.003067
**Gm6588**	-0.56	4.17E-05	0.003092
**4933425O20Rik**	-0.34	4.32E-05	0.003178
**R3hdm2**	0.19	4.34E-05	0.003178
**Ttc28**	-0.37	4.33E-05	0.003178
**Npepps**	0.12	4.40E-05	0.00321
**Gm12576**	-0.64	4.47E-05	0.003246
**Ssbp1**	0.15	4.48E-05	0.003246
**Kctd21**	0.18	4.56E-05	0.003268
**Tk1**	-0.40	4.56E-05	0.003268
**Tmem9b**	0.13	4.57E-05	0.003268
**Pitpna**	0.14	4.59E-05	0.00327
**Crb1**	0.58	4.66E-05	0.003311
**Nt5dc2**	-0.39	4.74E-05	0.003326
**Pfdn1**	0.14	4.73E-05	0.003326
**Vdac1**	0.10	4.73E-05	0.003326
**Ostc**	0.17	4.76E-05	0.003329
**Cmpk1**	0.20	4.79E-05	0.003339
**Mapre2**	0.09	4.86E-05	0.003369
**Apbb2**	-0.14	5.01E-05	0.00346
**Vta1**	0.13	5.05E-05	0.003476
**Pfkp**	0.15	5.22E-05	0.003581
**2210020M01Rik**	-0.47	5.24E-05	0.003585
**Tmub2**	0.14	5.26E-05	0.003585
**Tet2**	-0.20	5.36E-05	0.003629
**Ythdf1**	0.11	5.37E-05	0.003629
**Spdef**	-0.62	5.43E-05	0.003659
**Slc14a1**	-0.30	5.53E-05	0.003705

**Timm22**	0.14	5.54E-05	0.003705
**Golm1**	-0.24	5.66E-05	0.00377
**A930024N18Rik**	-0.41	5.70E-05	0.003771
**Muc6**	-0.52	5.70E-05	0.003771
**Siglec1**	-0.45	5.81E-05	0.003831
**Morf4l1**	0.15	5.97E-05	0.003889
**Proca1**	-0.24	5.94E-05	0.003889
**Ror1**	-0.58	5.96E-05	0.003889
**Lifr**	-0.27	6.01E-05	0.003908
**AC125486.1**	-0.54	6.30E-05	0.00401
**B3galnt1**	0.19	6.30E-05	0.00401
**Pde6h**	-0.46	6.27E-05	0.00401
**Polh**	-0.23	6.26E-05	0.00401
**Tnfrsf19**	0.20	6.27E-05	0.00401
**Tspan18**	-0.56	6.21E-05	0.00401
**Mff**	0.12	6.33E-05	0.004016
**Grinl1a**	0.11	6.43E-05	0.004061
**Tulp3**	-0.20	6.49E-05	0.004083
**Leprotl1**	0.14	6.62E-05	0.004153
**Gm11721**	-0.47	6.67E-05	0.00417
**Mkks**	0.21	6.76E-05	0.004211
**Plec**	-0.24	6.98E-05	0.00432
**Rbx1**	0.11	6.98E-05	0.00432
**Tgfbr3**	-0.27	7.09E-05	0.004376
**Nt5c1a**	-0.33	7.19E-05	0.004391
**Slc1a2**	-0.19	7.17E-05	0.004391
**Tbcd**	-0.10	7.19E-05	0.004391
**Calm2**	0.16	7.23E-05	0.004398
**Exd2**	0.18	7.42E-05	0.004502
**Lca5l**	-0.32	7.51E-05	0.004524
**Ywhab**	0.12	7.49E-05	0.004524
**6330527O06Rik**	0.36	7.55E-05	0.004533
**D430018E03Rik**	-0.42	7.62E-05	0.004533
**Plce1**	-0.43	7.62E-05	0.004533
**Vps24**	0.11	7.60E-05	0.004533
**BC031181**	0.11	7.69E-05	0.004547
**P2ry1**	-0.49	7.72E-05	0.004547
**Sema5a**	-0.28	7.70E-05	0.004547
**Sdccag8**	0.15	7.75E-05	0.004552
**A330049M08Rik**	-0.55	7.96E-05	0.004662

**Zfp365**	0.17	8.03E-05	0.004687
**Slmo2**	0.12	8.14E-05	0.004734
**Napg**	0.14	8.19E-05	0.004744
**Tor3a**	-0.16	8.21E-05	0.004744
**Ace**	-0.27	8.36E-05	0.004816
**Rec8**	-0.63	8.50E-05	0.004881
**Ppp2cb**	0.11	8.53E-05	0.004886
**Rc3h2**	-0.29	8.71E-05	0.00497
**Ppfia1**	-0.15	8.82E-05	0.00502
**Cyp3a13**	-0.64	8.87E-05	0.00503
**Rbm15**	-0.25	8.97E-05	0.005073
**Stk32a**	-0.46	9.11E-05	0.005136
**Bcs1l**	0.15	9.20E-05	0.00517
**4833412C05Rik**	0.60	9.40E-05	0.005172
**B430010I23Rik**	-0.67	9.37E-05	0.005172
**Dlx1**	0.19	9.24E-05	0.005172
**Gm16201**	-0.67	9.29E-05	0.005172
**Ict1**	0.16	9.38E-05	0.005172
**P2ry13**	0.22	9.32E-05	0.005172
**Ppp2r5c**	0.13	9.31E-05	0.005172
**Fkbp5**	-0.42	9.44E-05	0.00518
**Ccdc6**	-0.23	9.58E-05	0.005191
**Chac2**	0.26	9.53E-05	0.005191
**Hat1**	0.23	9.51E-05	0.005191
**Pkd2l2**	0.16	9.55E-05	0.005191
**Ube2j2**	0.11	9.68E-05	0.005229
**1700121F15Rik**	-0.36	9.74E-05	0.005246
**1500003O03Rik**	0.11	9.99E-05	0.005365
**Dctn3**	0.15	0.0001	0.005377
**Ccdc72**	0.19	0.000101	0.00539
**Trappc2**	0.23	0.000101	0.00539
**Eif1**	0.12	0.000102	0.005403
**Slc35f4**	0.17	0.000102	0.005403
**Bet1**	0.24	0.000103	0.005433
**Gm15478**	-0.52	0.000103	0.005433
**4933401P06Rik**	-0.55	0.000105	0.005494
**Eif2ak1**	0.11	0.000105	0.005494
**Ssh1**	-0.14	0.000105	0.005494
**Ttc13**	-0.12	0.000106	0.005523
**Arpc3**	0.14	0.000107	0.005529

**4831426I19Rik**	-0.43	0.000109	0.005615
**Agbl2**	-0.55	0.000109	0.005615
**Zbtb1**	-0.15	0.000109	0.005619
**Grin2a**	-0.33	0.00011	0.005625
**4921513D23Rik**	-0.18	0.000111	0.005651
**Hcfc1r1**	0.18	0.000111	0.005651
**Aldh3a1**	-0.49	0.000112	0.005691
**Fnip2**	-0.22	0.000112	0.005691
**Man2a2**	-0.12	0.000114	0.005748
**Dnahc9**	-0.51	0.000115	0.005829
**Naip1**	-0.50	0.000117	0.005864
**Fam168b**	0.07	0.000118	0.005893
**Zfp398**	-0.17	0.000118	0.005893
**Elk4**	-0.15	0.000119	0.005961
**Gm16183**	-0.32	0.000121	0.006002
**Prkacb**	0.18	0.000122	0.006039
**Atp6v0d1**	0.13	0.000123	0.006049
**Gm15835**	-0.46	0.000123	0.006049
**Morn1**	-0.25	0.000122	0.006049
**Slc2a4**	-0.49	0.000123	0.006049
**Fat1**	-0.38	0.000125	0.006118
**Tnxb**	-0.47	0.000125	0.006118
**1500011B03Rik**	0.14	0.000126	0.006119
**Gm16982**	-0.40	0.000127	0.006146
**Rabgef1**	0.15	0.000127	0.006146
**Lmo4**	0.28	0.000128	0.006206
**Amn1**	0.19	0.000129	0.006233
**AC123699.1**	-0.29	0.00013	0.00626
**Agt**	-0.37	0.000131	0.006274
**Gm12592**	-0.26	0.000132	0.006274
**Gm15892**	-0.51	0.000132	0.006274
**Svil**	-0.23	0.000132	0.006274
**Gm15651**	-0.62	0.000135	0.006382
**Gm16854**	-0.62	0.000135	0.006382
**Rpl7l1**	0.11	0.000136	0.006402
**Trp53inp2**	0.13	0.000137	0.006473
**Tulp4**	-0.16	0.000138	0.006473
**Ero1lb**	0.18	0.000138	0.006486
**Gm16626**	-0.46	0.000139	0.006487
**Ube2f**	0.12	0.000142	0.006602

**Gm15869**	-0.61	0.000142	0.006607
**Gm16534**	-0.59	0.000144	0.006687
**Arhgap25**	0.53	0.000145	0.006688
**Pam16**	0.14	0.000145	0.00669
**Gm16334**	-0.59	0.000146	0.006733
**B230319C09Rik**	-0.43	0.000148	0.006781
**1110007A13Rik**	-0.16	0.000153	0.007
**Gde1**	0.14	0.000154	0.007028
**Arhgef12**	-0.12	0.000157	0.007135
**Rnft2**	0.14	0.000157	0.007137
**Fam133b**	0.13	0.000157	0.007139
**Shisa6**	-0.53	0.000158	0.007144
**AA986860**	-0.30	0.00016	0.007201
**Cyp2d22**	-0.18	0.000161	0.007231
**Fanci**	-0.38	0.000161	0.007231
**4930404I05Rik**	-0.35	0.000163	0.007302
**Sept8**	0.16	0.000163	0.007302
**Dok5**	0.24	0.000164	0.00732
**5830444B04Rik**	-0.53	0.000165	0.007341
**A2m**	-0.62	0.000166	0.007342
**Sema3a**	0.36	0.000167	0.007385
**Golgb1**	-0.11	0.000168	0.00742
**Oplah**	-0.22	0.000169	0.007422
**C030046E11Rik**	-0.13	0.00017	0.007474
**AC122296.1**	-0.58	0.000171	0.007475
**Cyp2u1**	-0.20	0.000172	0.00752
**Dap3**	0.10	0.000177	0.007682
**Dnajc8**	0.11	0.000177	0.007682
**Gng11**	0.37	0.000177	0.007682
**Nsd1**	-0.16	0.000177	0.007682
**Rad54l**	-0.40	0.000178	0.007682
**AC163296.1**	-0.41	0.00018	0.007777
**Fam151b**	0.24	0.000181	0.007802
**Tmem65**	0.20	0.000182	0.007823
**Fbxw7**	0.18	0.000187	0.007968
**Gm14286**	-0.40	0.000187	0.007968
**Mdh1**	0.15	0.000187	0.007968
**Rab6**	0.21	0.000187	0.007968
**Nova2**	-0.19	0.000188	0.007988
**Slco1c1**	0.18	0.000191	0.008098

**Ppp2r2a**	0.14	0.000193	0.008138
**Dnaja1**	0.18	0.000196	0.008172
**F630040L22Rik**	-0.54	0.000196	0.008172
**Gm12216**	-0.53	0.000196	0.008172
**Nav2**	-0.18	0.000196	0.008172
**Nkiras1**	0.14	0.000195	0.008172
**Stmn2**	0.14	0.0002	0.008326
**BC060267**	-0.29	0.000201	0.008337
**Fscn2**	-0.49	0.000201	0.008337
**6530402F18Rik**	-0.24	0.000202	0.008357
**Dnaja3**	0.09	0.000203	0.008394
**Ehmt1**	-0.12	0.000205	0.008443
**Psma5**	0.13	0.000206	0.008443
**Mdk**	-0.26	0.000209	0.008535
**Tshb**	-0.62	0.000209	0.008535
**Ubl4**	0.11	0.000209	0.008535
**Zxdc**	-0.12	0.000211	0.008597
**C230096C10Rik**	-0.13	0.000213	0.008627
**Cpxm1**	-0.28	0.000213	0.008627
**Snap25**	0.14	0.000213	0.008627
**Idh3a**	0.14	0.000214	0.008655
**Cckbr**	0.29	0.000215	0.008657
**8030462N17Rik**	-0.18	0.000217	0.008737
**Sat1**	0.23	0.000219	0.008774
**Chst2**	-0.22	0.000222	0.008899
**AC148089.1**	-0.53	0.000223	0.008906
**Pde4b**	0.15	0.000224	0.008906
**2610001J05Rik**	0.15	0.000227	0.008983
**Als2cr4**	0.15	0.000228	0.008983
**Fndc4**	0.12	0.000227	0.008983
**Gcnt7**	-0.43	0.000227	0.008983
**Tead1**	-0.23	0.000229	0.009022
**Chd4**	-0.14	0.000232	0.00906
**Eef2k**	-0.16	0.000232	0.00906
**Kctd20**	0.09	0.000231	0.00906
**Ppp2r5a**	0.13	0.000232	0.00906
**Gpr161**	-0.56	0.000232	0.009061
**Bcas2**	0.10	0.000233	0.00907
**7SK.49**	-0.48	0.000239	0.009277
**Rbm47**	-0.52	0.00024	0.009302

**Tnfaip8l1**	0.21	0.000244	0.009411
**Wdr62**	-0.30	0.000245	0.00943
**Gm11827**	-0.63	0.000245	0.009432
**Uchl5**	0.19	0.000247	0.009501
**Gm16575**	-0.61	0.000251	0.009616
**Prkar2b**	0.19	0.000252	0.009625
**9830001H06Rik**	-0.40	0.000257	0.009775
**Gm711**	-0.46	0.000257	0.009775
**Zfyve26**	-0.12	0.000257	0.009775
**Itga11**	-0.37	0.000259	0.009832
**Acvr2b**	-0.27	0.000261	0.009887
**Mpeg1**	0.17	0.000262	0.009892
**Btrc**	0.09	0.000265	0.009896
**Ncrna00081**	0.15	0.000264	0.009896
**Nutf2**	0.13	0.000265	0.009896
**Prr5**	-0.42	0.000264	0.009896
**Sardh**	-0.22	0.000263	0.009896
**Zan**	-0.47	0.000265	0.009896
**Cyb5r4**	0.18	0.00027	0.010057
**Invs**	-0.12	0.000271	0.010057
**Ntrk3**	-0.20	0.000271	0.010057
**Actr1a**	0.10	0.000275	0.010173
**Atg12**	0.14	0.000281	0.010392
**Gm16896**	-0.44	0.000284	0.010464
**Gm14372**	-0.47	0.000286	0.010492
**Nsmce2**	0.14	0.000286	0.010492
**Slc16a12**	-0.36	0.000286	0.010492
**Gm15774**	-0.32	0.000288	0.010501
**Rab14**	0.15	0.000288	0.010501
**Trrap**	-0.14	0.000288	0.010501
**Dctn5**	0.10	0.000289	0.010517
**Sipa1l3**	-0.24	0.000292	0.01058
**Hsp90ab1**	0.10	0.000293	0.010603
**Mrps14**	0.15	0.000294	0.010625
**Pole**	-0.39	0.000295	0.010625
**Rab1**	0.13	0.0003	0.010789
**Csnk1e**	0.20	0.000301	0.010799
**Rabif**	0.12	0.000302	0.010831
**Tbck**	-0.23	0.000304	0.010876
**Vti1a**	0.09	0.000305	0.010878

**AC131761.1**	-0.30	0.000312	0.011082
**C130071C03Rik**	0.19	0.000311	0.011082
**9130011J15Rik**	0.10	0.000312	0.011095
**Yaf2**	0.14	0.000314	0.011142
**Mrps36**	0.22	0.000316	0.011189
**Il12a**	0.55	0.00032	0.01128
**D4Wsu53e**	0.13	0.000321	0.011301
**Iars**	0.10	0.000321	0.011301
**Pamr1**	0.36	0.000327	0.011437
**Pcmt1**	0.12	0.000326	0.011437
**Bag2**	0.17	0.000328	0.01147
**1700084C01Rik**	0.24	0.000329	0.011471
**N28178**	0.17	0.000329	0.011471
**Nap1l1**	0.15	0.000331	0.011514
**Pla1a**	-0.41	0.000333	0.011569
**Megf6**	-0.36	0.000336	0.011622
**AC137156.2**	-0.49	0.000337	0.011652
**Lmln**	-0.15	0.000338	0.011673
**Baalc**	0.13	0.00034	0.011722
**Syt13**	0.15	0.000342	0.011765
**Nynrin**	-0.25	0.000344	0.011812
**Ndufc1**	0.13	0.000346	0.01185
**Klhdc8a**	0.31	0.000348	0.011909
**Gm12227**	-0.54	0.000349	0.011914
**Atxn1**	-0.21	0.000354	0.011998
**Chordc1**	0.23	0.000354	0.011998
**Ndufa3**	0.17	0.000354	0.011998
**Pcp4**	0.28	0.000355	0.011998
**Tomm34**	0.12	0.000355	0.011998
**Gpr88**	0.34	0.00036	0.0121
**Myo7a**	-0.16	0.000359	0.0121
**Nif3l1**	0.12	0.000359	0.0121
**Dnaja2**	0.14	0.000371	0.012432
**Lyst**	-0.23	0.000371	0.012438
**Svep1**	-0.59	0.000373	0.01248
**Fam81a**	0.18	0.000374	0.012489
**Fbxl3**	0.15	0.000375	0.012489
**Cap2**	0.18	0.000382	0.012658
**Gm16316**	-0.52	0.000382	0.012658
**Igf2bp3**	-0.28	0.000382	0.012658

**Gm16738**	-0.32	0.000383	0.01267
**Cacng3**	0.20	0.000388	0.012807
**BC067074**	-0.42	0.000391	0.012869
**Pram1**	-0.30	0.000394	0.012945
**2900011O08Rik**	0.10	0.000397	0.013025
**Col18a1**	-0.54	0.000397	0.013026
**Atp6v1c1**	0.13	0.000401	0.013028
**Cfdp1**	0.13	0.0004	0.013028
**Dusp27**	-0.52	0.0004	0.013028
**Eif2b1**	0.10	0.0004	0.013028
**Sdhd**	0.09	0.000401	0.013028
**AC121121.1**	-0.27	0.000404	0.013051
**Ankrd61**	-0.32	0.000404	0.013051
**Bnip3l**	0.14	0.000404	0.013051
**Rab2b**	0.13	0.000405	0.013072
**Cnnm4**	-0.20	0.000409	0.013168
**5730403B10Rik**	0.08	0.00041	0.013183
**Mef2c**	0.28	0.000411	0.013202
**Bbs1**	0.10	0.000414	0.013258
**Bhlhe41**	-0.22	0.000416	0.013293
**Kcng2**	-0.55	0.000418	0.013347
**AC117232.2**	-0.36	0.000421	0.01335
**Cct2**	0.09	0.000421	0.01335
**Drd5**	-0.60	0.000421	0.01335
**Tpcn2**	-0.38	0.000419	0.01335
**B3galt5**	-0.35	0.000422	0.013361
**Cuta**	0.20	0.000423	0.013361
**Hdac9**	0.23	0.000424	0.013383
**Rasl11b**	0.29	0.000425	0.013383
**Mrpl21**	0.15	0.000426	0.013384
**5730455O13Rik**	0.21	0.000431	0.013526
**Dnahc17**	-0.32	0.000432	0.013543
**Khdrbs3**	0.21	0.000433	0.013547
**Tomm22**	0.11	0.000435	0.01357
**Capn11**	0.48	0.000438	0.013575
**Cyp4f18**	-0.49	0.000437	0.013575
**Mtap1b**	-0.21	0.000436	0.013575
**Ndufc2**	0.12	0.000438	0.013575
**Paip2**	0.13	0.000439	0.013575
**Ap3s1**	0.21	0.00044	0.013583

**Trp53bp2**	-0.18	0.000441	0.013608
**Dnmt3a**	-0.14	0.000442	0.013613
**Aloxe3**	-0.32	0.000453	0.01392
**Nudt4**	0.20	0.000454	0.01392
**Obscn**	-0.38	0.000455	0.013946
**Ctgf**	0.39	0.000457	0.013972
**AA408865**	-0.37	0.000461	0.014088
**Pgk1**	0.13	0.000466	0.01421
**Gm1698**	-0.60	0.000471	0.014346
**Tmem52**	-0.59	0.000475	0.014435
**4933423P22Rik**	-0.54	0.000481	0.014582
**Unc50**	0.11	0.000483	0.014624
**Ctbp2**	-0.34	0.000487	0.014687
**Rint1**	-0.13	0.000487	0.014687
**Znrf3**	-0.23	0.000488	0.014703
**Sec62**	0.13	0.00049	0.014753
**Gm11202**	-0.53	0.000492	0.014762
**Gm15965**	-0.57	0.000493	0.014768
**Mrpl42**	0.18	0.000493	0.014768
**C78339**	0.19	0.000497	0.014794
**Inpp4a**	-0.13	0.000496	0.014794
**Mrps27**	0.12	0.000496	0.014794
**Tdgf1**	-0.58	0.000501	0.014898
**Airn**	-0.53	0.000502	0.014902
**Lrp1**	-0.17	0.000505	0.014956
**Sfxn3**	0.17	0.000508	0.015024
**5830432E09Rik**	-0.57	0.000512	0.015125
**Atg4b**	0.07	0.000513	0.015127
**Pdcd5**	0.13	0.000514	0.015127
**Tmem49**	0.14	0.000514	0.015127
**Oxct1**	0.15	0.000518	0.015198
**6720456B07Rik**	0.12	0.000521	0.015223
**Klhl22**	0.07	0.000521	0.015223
**Sdhaf2**	0.10	0.00052	0.015223
**Cab39**	0.13	0.000525	0.015315
**Tert**	-0.29	0.000526	0.015323
**Itgb7**	-0.54	0.000529	0.015371
**Naa38**	-0.17	0.000532	0.015449
**Msh4**	-0.25	0.000533	0.015451
**Slc36a2**	-0.51	0.000536	0.015503

**Lrrtm3**	0.18	0.00054	0.01559
**Cnot8**	0.08	0.000544	0.015688
**Atp5g1**	0.12	0.000545	0.015691
**2310003L22Rik**	0.12	0.000549	0.015724
**9230117E06Rik**	-0.57	0.00055	0.015724
**Arfip2**	0.07	0.000552	0.015724
**Mt2**	-0.33	0.000548	0.015724
**Ndel1**	0.11	0.000553	0.015724
**Nphs2**	-0.48	0.000549	0.015724
**Pef1**	0.15	0.000552	0.015724
**Tnrc6c**	-0.12	0.000549	0.015724
**Gm10561**	0.25	0.000554	0.015726
**Slain1**	0.12	0.000555	0.015727
**Gm13110**	-0.30	0.000556	0.015731
**Dpy30**	0.14	0.000561	0.01585
**Gm14471**	-0.47	0.000566	0.015956
**Tdo2**	-0.59	0.000566	0.015956
**Arhgap21**	-0.13	0.00057	0.015962
**Farsb**	0.11	0.00057	0.015962
**Scamp5**	0.09	0.00057	0.015962
**Tbc1d4**	-0.40	0.000569	0.015962
**Mir494**	-0.58	0.000572	0.015983
**Trpv4**	-0.35	0.000572	0.015983
**Ankrd55**	-0.33	0.000576	0.01603
**Rabl2**	0.15	0.000577	0.01603
**Rtn4**	0.18	0.000576	0.01603
**Mpped2**	0.23	0.000581	0.016119
**Lamb3**	-0.47	0.000584	0.016183
**Ocel1**	0.14	0.000586	0.016211
**Fxyd7**	0.32	0.000589	0.016275
**Tceal8**	0.19	0.000598	0.016501
**Lrrc8a**	-0.21	0.000602	0.016585
**4930521C21Rik**	-0.38	0.000608	0.016701
**St18**	-0.57	0.000609	0.016701
**Ucp2**	-0.32	0.000608	0.016701
**Fbxo5**	0.40	0.000616	0.016864
**Cpne8**	0.21	0.000624	0.017019
**Ier3ip1**	0.16	0.000624	0.017019
**Stx1a**	0.25	0.000623	0.017019
**Ctnna3**	-0.53	0.000626	0.017031

**Cml2**	0.49	0.000632	0.01712
**Ebf3**	-0.52	0.00063	0.01712
**Mkrn1**	0.09	0.000633	0.01712
**Rala**	0.14	0.000631	0.01712
**Alpk1**	-0.24	0.000635	0.017123
**Spen**	-0.16	0.000635	0.017123
**Chst10**	0.14	0.000638	0.017159
**Igf2r**	-0.13	0.000639	0.017159
**Trim62**	-0.39	0.000638	0.017159
**Enkur**	0.35	0.000645	0.017288
**RP23-145O4.5**	-0.58	0.000649	0.017375
**Shmt1**	-0.28	0.00065	0.017375
**Tspan4**	-0.29	0.000651	0.017375
**Sh3gl2**	0.20	0.000654	0.017449
**2810025M15Rik**	0.20	0.000659	0.017547
**Ada**	-0.38	0.000671	0.017781
**Dlg1**	0.11	0.00067	0.017781
**Gm10564**	-0.40	0.000669	0.017781
**Il34**	0.20	0.000675	0.017861
**Wnt1**	-0.37	0.000678	0.01793
**Dnmbp**	-0.19	0.000684	0.018049
**Ppara**	-0.29	0.000686	0.018091
**Rufy3**	0.11	0.000688	0.018111
**Slc12a3**	-0.54	0.000692	0.018202
**Pion**	-0.20	0.000695	0.018229
**Tmem201**	-0.17	0.000698	0.018295
**Grb14**	0.26	0.000702	0.01836
**Fam164a**	0.19	0.000705	0.018394
**Fam192a**	0.09	0.000705	0.018394
**Tpm3**	0.11	0.000714	0.018612
**Aatf**	-0.13	0.000717	0.018655
**SNORA17.403**	-0.58	0.000718	0.018655
**Crtc3**	-0.16	0.000721	0.01872
**Fbxl7**	-0.42	0.000735	0.019037
**4921522P10Rik**	-0.50	0.000738	0.019062
**Arhgap31**	-0.19	0.000738	0.019062
**1110049F12Rik**	0.11	0.000743	0.019143
**Gm15816**	-0.42	0.000742	0.019143
**Ilf2**	0.09	0.00075	0.019283
**Stag3**	-0.36	0.00075	0.019283

**Rnf11**	0.18	0.000755	0.019359
**Txn1**	0.14	0.00076	0.01948
**Gm11750**	-0.58	0.000762	0.019484
**Lrrc20**	0.11	0.000768	0.019583
**Npas3**	-0.22	0.000767	0.019583
**Zc3h15**	0.16	0.000769	0.019586
**Heg1**	-0.20	0.000771	0.019607
**Cd27**	-0.51	0.000776	0.019713
**Map3k1**	-0.28	0.000781	0.019793
**Serinc3**	0.15	0.000781	0.019793
**Slc9a2**	-0.44	0.000783	0.019803
**Angptl7**	-0.47	0.000785	0.01983
**Angptl2**	-0.41	0.000791	0.019943
**Fam159b**	0.39	0.000791	0.019943
**Casc1**	-0.44	0.000798	0.020073
**Rtn3**	0.14	0.000801	0.020137
**Reep5**	0.13	0.000804	0.020173
**Gm15800**	-0.12	0.000807	0.0202
**Gm15813**	-0.40	0.000807	0.0202
**1700112E06Rik**	0.57	0.000812	0.020283
**Ostm1**	0.12	0.000815	0.020339
**Tusc3**	0.14	0.00082	0.020427
**2810006K23Rik**	0.19	0.000822	0.020452
**Slc22a12**	-0.52	0.000823	0.020466
**Ttl**	0.14	0.000825	0.020486
**Loxhd1**	-0.55	0.000833	0.020622
**Tmem2**	-0.20	0.000832	0.020622
**Klf6**	0.27	0.000837	0.020671
**Tmem55a**	0.11	0.000836	0.020671
**Fam40b**	-0.44	0.000844	0.020786
**Pappa**	-0.57	0.000844	0.020786
**Acr**	-0.35	0.000846	0.020795
**Ncam1**	-0.15	0.000847	0.020795
**3830431G21Rik**	-0.54	0.000859	0.021037
**M6pr**	0.10	0.000858	0.021037
**Slitrk6**	-0.53	0.000868	0.021223
**Pvrl4**	-0.35	0.000871	0.021246
**RP23-389D15.1**	-0.52	0.00087	0.021246
**Vapa**	0.14	0.000874	0.021256
**Zbtb42**	-0.48	0.000873	0.021256

**Mrpl9**	0.10	0.000877	0.021317
**Ccdc56**	0.15	0.000887	0.0215
**Fryl**	-0.15	0.000887	0.0215
**Rab15**	0.15	0.00089	0.021542
**Bex1**	0.19	0.000893	0.021591
**Dgkg**	-0.28	0.000899	0.021702
**Hnrnpa2b1**	0.13	0.000901	0.021725
**Ksr1**	-0.31	0.000906	0.021811
**Timp1**	-0.36	0.000911	0.021903
**Zcrb1**	0.14	0.000914	0.021936
**Bclp2**	-0.56	0.000918	0.021972
**Muc19**	-0.42	0.000917	0.021972
**Nlgn3**	-0.17	0.00092	0.021972
**Slc4a4**	-0.19	0.00092	0.021972
**Spats2l**	0.17	0.000923	0.022022
**Ryr1**	-0.33	0.000926	0.022043
**Tuba1a**	0.15	0.000926	0.022043
**Fank1**	-0.24	0.000928	0.022048
**Dlst**	0.08	0.000929	0.022053
**Rnf14**	0.15	0.000932	0.022077
**AC160104.2**	-0.25	0.000941	0.022278
**Foxm1**	-0.32	0.000946	0.022342
**Ubap1**	0.11	0.000946	0.022342
**Adamts18**	-0.56	0.000952	0.022365
**Ap3m2**	0.10	0.000957	0.022365
**Ep400**	-0.09	0.000949	0.022365
**Fxyd6**	0.18	0.000956	0.022365
**Gmps**	-0.23	0.000953	0.022365
**Lnx2**	-0.31	0.000957	0.022365
**Sema4b**	-0.23	0.000952	0.022365
**Zfp568**	-0.30	0.000956	0.022365
**Lrp4**	-0.23	0.00097	0.022636
**Hnrnpul2**	0.08	0.000972	0.022645
**Timm23**	0.09	0.000973	0.022645
**4933431E20Rik**	0.13	0.000976	0.022693
**Pdhb**	0.09	0.000988	0.022931
**Tmem44**	0.30	0.000989	0.022931
**C4b**	-0.30	0.000996	0.023034
**Gm14252**	-0.56	0.000996	0.023034
**Parva**	-0.15	0.000998	0.023056

**Ap2m1**	0.12	0.001	0.023062
**Stmn4**	0.18	0.001003	0.023103
**Spcs1**	0.11	0.001017	0.023418
**Gm16154**	-0.49	0.00103	0.023677
**A430105I19Rik**	-0.25	0.001039	0.023842
**Enoph1**	0.11	0.001041	0.023865
**Tmf1**	-0.16	0.001045	0.023923
**Cdc5l**	0.11	0.001048	0.023975
**Arsa**	0.10	0.001052	0.024037
**3110070M22Rik**	-0.43	0.001063	0.024182
**Gm11186**	-0.56	0.001063	0.024182
**Oat**	0.09	0.001062	0.024182
**Llph**	0.16	0.001065	0.024214
**6330577E15Rik**	0.14	0.001069	0.024258
**Odf2**	-0.11	0.001072	0.024294
**4930578G10Rik**	-0.40	0.001081	0.024475
**Batf3**	-0.47	0.001083	0.024475
**Cldn12**	0.15	0.001083	0.024475
**Atic**	0.12	0.001086	0.024478
**Thy1**	0.12	0.001086	0.024478
**4933427I04Rik**	-0.20	0.001091	0.024523
**A830093I24Rik**	-0.25	0.001092	0.024523
**Fahd1**	0.15	0.001092	0.024523
**AW011738**	-0.26	0.001096	0.024537
**Cacna1h**	-0.27	0.001099	0.024537
**Gm14376**	-0.55	0.001099	0.024537
**Mobkl2b**	-0.32	0.001097	0.024537
**Txnrd3**	-0.29	0.0011	0.024537
**Gm16197**	-0.52	0.001104	0.024615
**Basp1**	0.16	0.001107	0.024647
**Cyp4v3**	-0.19	0.001123	0.024885
**Gm14397**	-0.56	0.001122	0.024885
**Gm16907**	-0.32	0.001122	0.024885
**Usp29**	-0.21	0.001123	0.024885
**Snrpa**	0.13	0.001129	0.02497
**Tram1l1**	0.12	0.001133	0.025008
**Yars**	0.10	0.001132	0.025008
**Fam115a**	0.13	0.001136	0.025042
**Sdc1**	-0.53	0.001137	0.025042
**1300010F03Rik**	-0.13	0.001144	0.025162

**Rgag1**	-0.29	0.001147	0.025193
**Ivns1abp**	0.14	0.001148	0.025195
**Bsn**	-0.19	0.001152	0.025243
**Gm15863**	-0.38	0.001153	0.025243
**Gm15265**	-0.29	0.00116	0.025353
**Capns2**	-0.38	0.001168	0.025501
**Sybu**	0.09	0.001169	0.025501
**Gng5**	0.13	0.001178	0.025663
**Adam22**	-0.16	0.00118	0.025664
**Pdia3**	0.15	0.001194	0.025944
**Kif3a**	0.10	0.0012	0.026007
**Map6d1**	0.15	0.001199	0.026007
**Amica1**	-0.55	0.001207	0.026051
**Ptprk**	0.19	0.001204	0.026051
**Scarna9**	-0.48	0.001206	0.026051
**Xdh**	-0.31	0.001206	0.026051
**Dnase2a**	-0.19	0.001218	0.026137
**Hrh1**	0.23	0.001217	0.026137
**Ints3**	0.09	0.001216	0.026137
**Mir486**	-0.55	0.001217	0.026137
**Telo2**	-0.16	0.001218	0.026137
**Golga7**	0.11	0.001225	0.026247
**Rnf180**	0.17	0.001228	0.02628
**Abhd6**	0.14	0.001232	0.026338
**Ensa**	0.11	0.001238	0.026429
**Kdm2b**	-0.15	0.001244	0.026536
**Fry**	-0.13	0.001248	0.026568
**Gm7292**	-0.16	0.001249	0.026568
**Kdm5b**	-0.11	0.001261	0.026792
**4930594M22Rik**	-0.45	0.001263	0.026802
**Gm11681**	-0.43	0.001265	0.026812
**Gm10390**	-0.52	0.001271	0.026924
**Gabre**	-0.49	0.001277	0.027016
**St3gal1**	0.23	0.001287	0.027187
**4631416L12Rik**	-0.17	0.001292	0.027196
**Prom2**	-0.54	0.001291	0.027196
**Supt4h1**	0.14	0.00129	0.027196
**Asns**	0.11	0.001294	0.02722
**Txndc9**	0.10	0.001297	0.02725
**Rab5b**	0.07	0.001308	0.02744

**Ngfr**	-0.47	0.001319	0.027643
**Them4**	0.18	0.001321	0.027667
**Akr1b3**	0.12	0.001324	0.027699
**Nfib**	-0.25	0.001326	0.027701
**A3galt2**	-0.52	0.001343	0.027902
**Cmpk2**	0.23	0.001343	0.027902
**Lgmn**	0.17	0.001341	0.027902
**Lpin3**	-0.49	0.001342	0.027902
**Rps6ka5**	-0.23	0.001343	0.027902
**Gm15608**	-0.34	0.001349	0.027948
**Myo1e**	0.27	0.001347	0.027948
**9030617O03Rik**	-0.18	0.001356	0.028009
**Shank1**	-0.29	0.001354	0.028009
**Uap1**	0.18	0.001357	0.028009
**Usp54**	-0.18	0.001358	0.028009
**Hmgn3**	0.11	0.001362	0.028032
**Rc3h1**	-0.19	0.001361	0.028032
**Atp6v0b**	0.14	0.001363	0.028033
**Hmg20a**	0.10	0.001375	0.028204
**Lct**	-0.47	0.001374	0.028204
**Gm12977**	-0.54	0.001383	0.028303
**Myst3**	-0.13	0.001383	0.028303
**Fubp1**	0.15	0.001387	0.028321
**Trib2**	0.28	0.001385	0.028321
**2810004N23Rik**	0.15	0.001391	0.028376
**4930556I23Rik**	-0.36	0.001402	0.028516
**Cyb561**	0.13	0.0014	0.028516
**Entpd7**	-0.17	0.001402	0.028516
**Gm5901**	-0.17	0.001408	0.028552
**Slc22a4**	-0.33	0.001409	0.028552
**Zc3hav1**	-0.22	0.001409	0.028552
**Pde6b**	-0.51	0.001411	0.028554
**Copz1**	0.10	0.001415	0.028604
**Rnf181**	0.10	0.001416	0.028604
**Smad9**	-0.18	0.001425	0.028751
**Eif4e**	0.14	0.001431	0.028839
**Irgm1**	0.22	0.001443	0.029052
**Gchfr**	-0.53	0.001448	0.029124
**Git2**	-0.18	0.001456	0.029247
**Gm11126**	-0.32	0.001458	0.029247

**Enah**	-0.15	0.00146	0.029271
**Dusp14**	0.22	0.001477	0.029572
**7SK.5**	0.54	0.001488	0.029755
**1810063B07Rik**	0.17	0.001494	0.029845
**Sec16a**	-0.09	0.001495	0.029845
**Ceacam2**	-0.44	0.001503	0.029901
**Hprt**	0.15	0.001503	0.029901
**Qrsl1**	0.14	0.001503	0.029901
**Hnf1b**	-0.50	0.001508	0.029903
**Mrpl49**	0.10	0.001508	0.029903
**Snap47**	0.10	0.001507	0.029903
**3110035E14Rik**	0.32	0.001515	0.030007
**Arl1**	0.12	0.001523	0.030093
**Snx15**	0.15	0.001522	0.030093
**Acsf2**	-0.14	0.001529	0.030127
**Atg7**	0.11	0.001529	0.030127
**Tnrc18**	-0.21	0.001528	0.030127
**9130019P16Rik**	-0.49	0.001545	0.030394
**Atpaf1**	0.13	0.00155	0.030406
**Ghitm**	0.11	0.001549	0.030406
**Kif26b**	-0.30	0.001549	0.030406
**Apoe**	0.16	0.001554	0.030441
**Neb**	-0.44	0.001561	0.03055
**Mboat2**	-0.16	0.001567	0.030638
**Morf4l2**	0.14	0.001571	0.030692
**Arpp21**	0.15	0.00158	0.030808
**Creld1**	0.17	0.001581	0.030808
**9330154K18Rik**	-0.52	0.001588	0.030878
**Fzd7**	-0.34	0.001591	0.030878
**Mrpl19**	0.12	0.001589	0.030878
**Nr3c2**	-0.26	0.001591	0.030878
**Sult5a1**	-0.53	0.001596	0.030907
**Tomm5**	0.15	0.001595	0.030907
**Gm15631**	0.25	0.0016	0.030954
**Extl2**	0.18	0.001603	0.030971
**Gemin8**	0.13	0.001608	0.030985
**Nexn**	-0.44	0.001608	0.030985
**Tbc1d16**	-0.18	0.001607	0.030985
**Abcc12**	-0.54	0.001615	0.031076
**Psenen**	0.14	0.001619	0.031114

**Mzt1**	0.18	0.001624	0.031126
**Pdp2**	-0.19	0.001622	0.031126
**Tmem107**	0.24	0.001623	0.031126
**Gm16601**	-0.26	0.001633	0.031261
**Klhl3**	-0.22	0.001642	0.03131
**Pofut1**	-0.14	0.001638	0.03131
**Qrfp**	-0.53	0.001639	0.03131
**Trpa1**	-0.54	0.001642	0.03131
**P2ry12**	0.17	0.001645	0.031318
**Slc9a3**	-0.53	0.00165	0.03138
**4930547N16Rik**	-0.46	0.001654	0.031405
**Pdzd11**	0.11	0.001653	0.031405
**Chst15**	-0.10	0.001661	0.031419
**Gm1987**	-0.54	0.001665	0.031419
**Mmachc**	0.14	0.001659	0.031419
**Pip4k2b**	0.11	0.001661	0.031419
**Tbr1**	0.25	0.001663	0.031419
**Tmem50b**	0.11	0.001665	0.031419
**Fam83d**	-0.53	0.001681	0.03169
**Hexdc**	-0.10	0.001691	0.031808
**Igf1r**	-0.18	0.00169	0.031808
**Ndufa4**	0.13	0.001694	0.031833
**2510003E04Rik**	0.07	0.001699	0.031882
**4930422G04Rik**	-0.23	0.001712	0.032077
**Snrpb2**	0.18	0.001712	0.032077
**Cycs**	0.14	0.001727	0.032324
**Comp**	-0.54	0.001731	0.032359
**7SK.312**	-0.54	0.001737	0.032443
**R3hdm1**	0.14	0.001746	0.032565
**Ppid**	0.13	0.001749	0.032597
**Pknox2**	0.15	0.001752	0.032623
**Olfm2**	0.32	0.001756	0.03266
**Kctd8**	-0.36	0.001776	0.032786
**Mcts1**	0.16	0.001775	0.032786
**Nos1**	-0.31	0.001777	0.032786
**Pitpnm2**	-0.23	0.001774	0.032786
**Smarcd2**	-0.22	0.001773	0.032786
**Sod3**	-0.22	0.001768	0.032786
**Tardbp**	0.15	0.00177	0.032786
**Tex11**	-0.51	0.001771	0.032786

**Mtmr6**	0.13	0.001782	0.032809
**Stk39**	0.13	0.001781	0.032809
**4930534B04Rik**	-0.20	0.001788	0.032843
**AC118017.1**	-0.29	0.001798	0.032843
**Barx2**	0.32	0.0018	0.032843
**Dimt1**	0.14	0.001794	0.032843
**Elavl4**	0.24	0.00179	0.032843
**Gpr12**	0.23	0.001789	0.032843
**Ltbp3**	-0.26	0.001795	0.032843
**Mapk10**	0.13	0.001798	0.032843
**Sfrp1**	0.33	0.001798	0.032843
**Gm15787**	-0.30	0.001805	0.032895
**AC044864.2**	-0.20	0.00181	0.032934
**Klf13**	-0.21	0.001809	0.032934
**Dazap2**	0.09	0.001814	0.03297
**Wnk4**	-0.32	0.001821	0.033057
**Col17a1**	-0.53	0.001827	0.033134
**Megf11**	-0.33	0.00183	0.033169
**Necap1**	0.11	0.001837	0.033254
**Alkbh5**	-0.15	0.001842	0.033279
**Atp6v1d**	0.11	0.001841	0.033279
**Uba3**	0.18	0.001848	0.033347
**Cdc123**	0.09	0.001855	0.033422
**Wnt5b**	-0.37	0.001855	0.033422
**Man2b2**	-0.17	0.001861	0.033494
**Txndc15**	0.09	0.001866	0.033539
**Cpt2**	-0.18	0.001879	0.033742
**SNORA17.514**	-0.51	0.001883	0.033784
**Atp6ap2**	0.16	0.001888	0.033826
**Tfg**	0.10	0.001889	0.033826
**Gm10925**	0.25	0.001891	0.033827
**Gm13405**	-0.53	0.001898	0.033923
**Arntl**	0.19	0.001909	0.034
**Chn1**	0.10	0.001912	0.034
**Dctn6**	0.10	0.001911	0.034
**Hsd17b12**	0.11	0.001905	0.034
**Mras**	0.11	0.001909	0.034
**Tmem194**	-0.21	0.001917	0.034065
**Aanat**	-0.32	0.001922	0.034125
**Pard6g**	-0.26	0.001933	0.034288

**Grin3a**	-0.15	0.001945	0.034466
**Atl1**	0.11	0.001949	0.034475
**Nxn**	-0.26	0.001951	0.034475
**Uros**	0.11	0.001951	0.034475
**Rbmxrt**	0.11	0.001954	0.034478
**A430071A18Rik**	-0.52	0.001959	0.034547
**Golph3**	0.12	0.001968	0.034669
**Add1**	0.08	0.001974	0.034737
**Fam107b**	-0.47	0.001976	0.034737
**Tusc2**	0.12	0.001992	0.034991
**AL929166.1**	0.25	0.001996	0.035033
**Gm13069**	-0.47	0.001999	0.03504
**Ppp3r1**	0.16	0.002011	0.035221
**Kcnk10**	-0.33	0.002017	0.035296
**9330162012Rik**	-0.24	0.00202	0.035312
**1700015E13Rik**	-0.45	0.002024	0.035342
**H2-M10.2**	-0.29	0.002042	0.035622
**Rps6ka1**	-0.35	0.002044	0.035622
**Chsy3**	0.25	0.002048	0.035626
**Gca**	0.18	0.002047	0.035626
**Fam174a**	0.12	0.002051	0.035641
**Ndufv2**	0.12	0.002062	0.035801
**Cerkl**	-0.37	0.002069	0.035889
**1810063B05Rik**	0.18	0.002076	0.035911
**Gm15722**	-0.53	0.002075	0.035911
**Tekt4**	-0.53	0.002075	0.035911
**Gm16952**	-0.47	0.002078	0.035917
**Ddo**	-0.29	0.002084	0.035984
**L1cam**	0.12	0.002093	0.0361
**Kit**	-0.27	0.0021	0.036198
**Otx1**	0.37	0.002112	0.036346
**Sv2c**	-0.20	0.002113	0.036346
**2310040G07Rik**	-0.43	0.002117	0.036375
**Cadps2**	-0.38	0.002123	0.036389
**Gm11201**	-0.47	0.002123	0.036389
**Zwint**	0.12	0.002122	0.036389
**Ppargc1b**	-0.24	0.002126	0.036394
**B3gat1**	0.13	0.002143	0.036622
**Dpf3**	-0.47	0.002142	0.036622
**Rere**	-0.13	0.002147	0.03665

**Atp1a4**	-0.44	0.002158	0.036817
**Itpkb**	-0.23	0.002164	0.036855
**Slc6a5**	-0.42	0.002165	0.036855
**Zranb2**	0.18	0.002169	0.036894
**Kif3b**	0.09	0.002179	0.037026
**AC163094.1**	0.25	0.002208	0.037478
**Npb**	-0.52	0.002209	0.037478
**Echdc2**	-0.27	0.002221	0.03758
**Gatad2b**	-0.17	0.002225	0.03758
**Mrpl40**	0.15	0.002226	0.03758
**Stk11ip**	0.14	0.002218	0.03758
**Zfp111**	-0.12	0.002226	0.03758
**Fam123c**	-0.15	0.002237	0.037732
**Ttc36**	-0.50	0.002251	0.037934
**Gatc**	0.10	0.002261	0.038043
**Per3**	-0.31	0.00226	0.038043
**Bptf**	-0.11	0.002267	0.038095
**Fkbp3**	0.16	0.002274	0.038125
**Olfr544**	-0.52	0.002277	0.038125
**Spns3**	-0.42	0.002275	0.038125
**Vps4a**	0.08	0.002272	0.038125
**1110002B05Rik**	0.14	0.002283	0.038204
**Slc22a18**	-0.39	0.002287	0.038222
**Cpe**	0.11	0.002297	0.038357
**1110008P14Rik**	0.31	0.002303	0.038396
**A930001C03Rik**	-0.52	0.002303	0.038396
**Uck1**	0.11	0.002311	0.038486
**Pxn**	-0.26	0.002313	0.038486
**Gm11738**	-0.52	0.002321	0.038582
**Klhl18**	0.11	0.002331	0.038683
**Lamb2**	-0.24	0.002331	0.038683
**Clmn**	-0.17	0.002336	0.03871
**Pcdha1**	0.12	0.002337	0.03871
**Rbm17**	0.08	0.00234	0.038732
**Fat4**	-0.28	0.002344	0.038754
**Phyhipl**	0.11	0.002347	0.038773
**Stard3nl**	0.11	0.002354	0.038817
**Wls**	0.22	0.002353	0.038817
**Cebpg**	0.14	0.002359	0.038871
**Egr1**	0.47	0.00238	0.039144

**Siah1a**	0.11	0.00238	0.039144
**Prr12**	-0.22	0.002387	0.039227
**Slc22a15**	-0.22	0.00239	0.039227
**Sgtb**	0.19	0.002399	0.03935
**2310046A06Rik**	0.17	0.002417	0.03951
**B3gat2**	-0.16	0.002415	0.03951
**Gm9754**	-0.44	0.002419	0.03951
**Nfya**	0.17	0.002424	0.03951
**P2rx1**	-0.50	0.002423	0.03951
**Pcsk2**	0.15	0.00242	0.03951
**Wnt6**	-0.45	0.002424	0.03951
**C1ql2**	-0.52	0.002429	0.039521
**Pskh1**	-0.14	0.002429	0.039521
**AI480653**	0.15	0.002441	0.039672
**Commd3**	0.11	0.002459	0.039943
**Lyrm1**	0.14	0.002463	0.039959
**Prx**	-0.32	0.002487	0.040292
**Tmem127**	0.09	0.002486	0.040292
**Vps45**	0.11	0.00249	0.040294
**Srsf6**	0.11	0.002495	0.040345
**Fkbp10**	-0.22	0.002519	0.040656
**Sox2**	0.21	0.002518	0.040656
**Cd163**	-0.39	0.00253	0.040751
**Gm16267**	-0.37	0.002531	0.040751
**Tmem170**	-0.27	0.002531	0.040751
**Cdc42**	0.14	0.002544	0.040877
**Hdac4**	-0.16	0.002542	0.040877
**2010002N04Rik**	-0.33	0.002555	0.040932
**Alms1**	-0.25	0.002552	0.040932
**Tra2a**	0.17	0.002556	0.040932
**Znrf1**	0.14	0.002551	0.040932
**Ghrl**	-0.23	0.002566	0.041023
**Sycp2**	-0.28	0.002566	0.041023
**Rtp1**	-0.51	0.002582	0.041232
**Ntn1**	-0.34	0.002586	0.041267
**H2afz**	0.11	0.002602	0.041449
**Prrt4**	-0.31	0.002601	0.041449
**Glrx5**	0.15	0.002607	0.041501
**Arf2**	0.10	0.002631	0.041514
**BC018473**	-0.44	0.002618	0.041514

**C130039O16Rik**	-0.13	0.002614	0.041514
**Cldn1**	0.38	0.002628	0.041514
**Gm15345**	-0.50	0.002629	0.041514
**Kifc2**	0.16	0.002616	0.041514
**Mpp4**	-0.41	0.002621	0.041514
**Rac1**	0.09	0.002619	0.041514
**Snx3**	0.11	0.002629	0.041514
**Wwc2**	-0.12	0.002627	0.041514
**Fam136a**	0.14	0.002635	0.041546
**Cdc42bpg**	-0.37	0.002661	0.041888
**Gas2l3**	-0.31	0.002664	0.041888
**Tspyl1**	0.08	0.002662	0.041888
**RP23-353F16.2**	-0.52	0.002668	0.04192
**Psap**	0.09	0.002686	0.042132
**Serpine1**	-0.43	0.002686	0.042132
**Mrpl50**	0.14	0.002694	0.042223
**Zic2**	-0.48	0.002704	0.042344
**Reep1**	0.13	0.002721	0.042562
**Pfn2**	0.12	0.002731	0.04269
**Ebna1bp2**	0.09	0.002735	0.042717
**Gtf2h5**	0.10	0.002742	0.042785
**Pfas**	-0.13	0.002754	0.042863
**Rorc**	-0.36	0.002754	0.042863
**Sgsh**	-0.17	0.002751	0.042863
**Adamts14**	-0.51	0.002761	0.042897
**Polg2**	-0.23	0.00276	0.042897
**Dhtkd1**	-0.15	0.002766	0.042913
**Dsg2**	0.34	0.002765	0.042913
**Plcg2**	-0.22	0.00277	0.042939
**Zdbf2**	-0.30	0.002781	0.043069
**Fndc1**	-0.30	0.002788	0.043137
**Rap1gap2**	0.11	0.002797	0.043241
**Hexa**	0.13	0.002804	0.043308
**Dopey2**	-0.10	0.002811	0.043347
**Mapk8**	0.23	0.00281	0.043347
**Rab9**	0.10	0.002826	0.043536
**1700061G19Rik**	-0.40	0.002831	0.043579
**Syce2**	-0.25	0.002835	0.043612
**6430562O15Rik**	-0.32	0.002843	0.043653
**Pcna**	0.09	0.002843	0.043653

**A930028C08Rik**	-0.32	0.002851	0.043742
**4921518J05Rik**	-0.27	0.002858	0.043816
**Atg2b**	-0.11	0.002863	0.043849
**Cox7b**	0.13	0.002868	0.043892
**1700056N10Rik**	-0.22	0.002888	0.044118
**Acyp1**	0.20	0.00289	0.044118
**Gm14015**	0.51	0.002888	0.044118
**Hnrnpm**	0.11	0.002893	0.044128
**Glt8d2**	0.24	0.002897	0.044157
**Atp2a3**	-0.49	0.002901	0.044176
**Bloc1s3**	0.16	0.002904	0.044186
**Mterfd1**	0.15	0.002923	0.044433
**Dusp22**	0.10	0.002931	0.044527
**Gm14169**	0.17	0.00295	0.044777
**Slc6a7**	0.15	0.002959	0.044872
**Ccdc78**	-0.37	0.002962	0.044889
**Akr1b8**	-0.37	0.002965	0.04489
**Ribc1**	-0.37	0.00297	0.044926
**AC151908.1**	-0.17	0.002977	0.044996
**Elmod3**	-0.13	0.002982	0.045041
**Gm10818**	-0.35	0.002992	0.045115
**Ston1**	-0.27	0.00299	0.045115
**Mtor**	-0.10	0.003011	0.045302
**Nhsl2**	-0.13	0.003012	0.045302
**Pyroxd2**	-0.25	0.003011	0.045302
**1700026J04Rik**	-0.34	0.003026	0.04548
**Uts2**	-0.51	0.003038	0.045625
**Bbs12**	0.23	0.003055	0.045756
**Cacng8**	-0.28	0.003054	0.045756
**P2rx5**	-0.44	0.00305	0.045756
**Cdca7l**	0.28	0.003062	0.045825
**Bzw1**	0.16	0.003076	0.045998
**Btg2**	0.37	0.003092	0.046198
**1700094C10Rik**	-0.51	0.003095	0.046203
**Plxnb1**	-0.16	0.003101	0.046203
**Prrg3**	0.13	0.003102	0.046203
**Vps29**	0.13	0.003098	0.046203
**Gm16719**	-0.51	0.003109	0.046234
**Tcp11**	-0.40	0.003108	0.046234
**Mkl2**	-0.14	0.003115	0.046278

**Mir665**	-0.48	0.003123	0.046362
**Dhdpsl**	-0.35	0.003127	0.046385
**Ccdc67**	-0.46	0.003137	0.046493
**4921536K21Rik**	-0.43	0.003149	0.046559
**Gm5077**	-0.40	0.003149	0.046559
**Tns1**	-0.17	0.003146	0.046559
**Nptn**	0.11	0.003174	0.046897
**Atp2c2**	-0.41	0.003185	0.046927
**Pqlc1**	-0.18	0.003187	0.046927
**Tmem177**	0.14	0.00318	0.046927
**Zcchc18**	0.11	0.003182	0.046927
**Tpo**	-0.50	0.00319	0.04694
**Tead4**	-0.50	0.003196	0.046989
**Hinfp**	-0.14	0.003212	0.047186
**Tcf25**	0.09	0.003223	0.04731
**Mat2b**	0.14	0.003227	0.047317
**St3gal6**	0.13	0.003229	0.047317
**Xrcc3**	-0.21	0.003235	0.047381
**Snrpd1**	0.16	0.003248	0.047529
**Cybasc3**	0.15	0.003252	0.047541
**Syn2**	0.09	0.003283	0.047962
**9130017K11Rik**	0.49	0.003292	0.048037
**Elp3**	0.08	0.003296	0.048037
**Mov10**	-0.22	0.003296	0.048037
**Abhd2**	-0.17	0.003311	0.048222
**Wbp4**	0.09	0.003318	0.048276
**Ccdc25**	0.14	0.003323	0.048282
**Gpd1l**	0.09	0.003325	0.048282
**Slc25a4**	0.08	0.003326	0.048282
**Kdm1a**	0.07	0.003331	0.048319
**Dffa**	0.09	0.00334	0.048376
**Ftsjd2**	0.10	0.00334	0.048376
**4930443O20Rik**	-0.49	0.003366	0.048568
**Aurkc**	-0.49	0.003359	0.048568
**Fgfr1op2**	0.15	0.003361	0.048568
**Mir1942**	-0.50	0.003367	0.048568
**Trem3**	0.50	0.003366	0.048568
**Dcxr**	-0.25	0.003372	0.048608
**Bnip3**	0.18	0.003382	0.048625
**Dusp6**	0.36	0.003384	0.048625

**Nphs1as**	-0.50	0.003378	0.048625
**Wnt9b**	-0.49	0.003383	0.048625
**Arhgap17**	-0.13	0.003389	0.048656
**Ank1**	-0.31	0.003399	0.048726
**Cep164**	-0.14	0.003399	0.048726
**Gon4l**	-0.06	0.003416	0.04893
**Osbpl1a**	0.23	0.003419	0.04894
**Abca2**	-0.14	0.003423	0.048958
**Ppp2r3c**	0.16	0.003431	0.049028
**Gm15489**	-0.32	0.003438	0.049093
**Shisa5**	0.15	0.003441	0.049093
**Abcc5**	0.13	0.00345	0.049177
**Gm10565**	-0.48	0.003452	0.049177
**CT030259.3**	-0.31	0.003455	0.049179
**BB114351**	-0.41	0.003465	0.04929
**Colq**	-0.50	0.003478	0.049394
**Tnrc6b**	-0.13	0.003478	0.049394
**Actb**	-0.11	0.003515	0.049877

Table S2. Differentially expressed genes from the PVC of *APOE3/4* vs. *APOE3/3* mice

Gene Name	log2 FoldChange	p-value	FDR
Serpina3m	1.86	1.00E-36	1.66E-32
Oscar	1.60	5.49E-29	4.55E-25
Serpina3n	1.53	4.45E-28	2.46E-24
Rdh13	-0.53	1.91E-26	7.90E-23
Ptprh	1.13	1.84E-14	6.09E-11
Thnsl1	0.49	3.19E-12	8.82E-09
Tmc4	0.71	5.55E-11	1.31E-07
Dctd	-0.57	9.84E-11	2.04E-07
Wdfy1	-0.54	1.12E-09	2.07E-06
Zc3h7b	0.25	2.11E-09	3.49E-06
Gm15494	-0.81	2.63E-09	3.96E-06
BC060267	-0.46	5.93E-09	8.18E-06
Fmo2	-0.77	1.96E-08	2.49E-05
Gm8759	0.48	2.29E-08	2.71E-05
2210404J11Rik	-0.31	3.78E-08	4.17E-05
Serpina3g	0.74	1.63E-07	0.000168
Asprv1	-0.62	4.73E-07	0.00046
RP24-161J8.2	-0.62	7.13E-07	0.000656
Gm1082	0.72	8.29E-07	0.000657
Hif3a	-0.67	8.33E-07	0.000657
Psg16	-0.23	7.59E-07	0.000657
Fbxo5	0.59	9.36E-07	0.000705
Ica1	-0.35	1.46E-06	0.001051
Maml3	0.46	5.88E-06	0.003894
Nrbp2	-0.26	5.76E-06	0.003894
1700048O20Rik	-0.46	9.66E-06	0.005928
Gm10931	-0.59	9.33E-06	0.005928
Gm1078	-0.46	1.30E-05	0.007719
Gm15594	-0.64	1.43E-05	0.008191
Pisd	-0.28	1.63E-05	0.009001
Gm16854	-0.61	1.77E-05	0.009452
Rps14	0.24	1.94E-05	0.010054
Gm5506	0.39	2.05E-05	0.010293
Gm10094	0.57	2.13E-05	0.010379
Rpa3	-0.34	2.53E-05	0.011949
C8g	-0.35	3.31E-05	0.014492
Gm10654	-0.40	3.32E-05	0.014492

**Rpl30**	0.20	3.16E-05	0.014492
**Cdkn2c**	0.45	4.27E-05	0.018126
**Akt2**	0.27	4.82E-05	0.019952
**Tcea2**	-0.23	4.96E-05	0.020029
**Gm9846**	0.45	5.45E-05	0.021494
**Mtmr4**	-0.16	7.52E-05	0.028961
**Zfp629**	-0.13	8.44E-05	0.031786
**Irak1**	-0.19	9.10E-05	0.03349
**Srp54b**	0.27	0.000108	0.039036
**Gm10268**	0.41	0.000122	0.042921
**Nhlh1**	-0.56	0.000131	0.044114
**Otud1**	0.28	0.000129	0.044114
**2500003M10Rik**	0.13	0.000133	0.044169
**Gm12394**	-0.46	0.000139	0.045173
**Rps3a**	0.19	0.000144	0.045888
**Large**	0.18	0.000158	0.048456
**Nudt3**	0.18	0.000158	0.048456
**Snrpd3**	0.21	0.000161	0.048456
